# Vertical eDNA distribution of cold‐water fishes in response to environmental variables in stratified lake

**DOI:** 10.1002/ece3.11091

**Published:** 2024-03-18

**Authors:** Kayoko Fukumori, Natsuko I. Kondo, Ayato Kohzu, Kenji Tsuchiya, Hiroshi Ito, Taku Kadoya

**Affiliations:** ^1^ Biodiversity Division National Institute for Environmental Studies (NIES) Ibaraki Japan; ^2^ Regional Environment Conservation Division National Institute for Environmental Studies (NIES) Ibaraki Japan

**Keywords:** eDNA, metabarcoding, salmon, species detection, stratification

## Abstract

In summer, the survival zones of cold‐water species are predicted to narrow by both increasing water temperatures from the surface and by expanding hypoxic zones from the lake bottom. To examine how the abundance of cold‐water fishes changes along environmental gradients, we assessed the vertical environmental DNA (eDNA) distributions of three salmonid species which may have different water temperature tolerances during both stratification and turnover periods using quantitative PCR (qPCR). In addition, we examined on the vertical distribution of diverse fish fauna using an eDNA metabarcoding assay. The results suggested that the kokanee salmon (*Oncorhynchus nerka*) eDNA were abundant in deep, cold waters. On the other hand, rainbow trout (*O. mykiss*) eDNA were distributed uniformly throughout the water column, suggesting that they may have high water‐temperature tolerance compared with kokanee salmon. The eDNA concentrations of masu salmon (*O. masou*) were below the detection limit (i.e., <10 copies μL^−1^) at all stations and depths and hence could not be quantified during stratification. Together with the finding that the eDNA distributions of other prey fish species were also constrained vertically in species‐specific ways, our results suggest that climate change will result in substantial changes in the vertical distributions of lake fish species and thus affect their populations and interactions.

## Graphical abstract

Vertical eDNA distribution of lake fishes.

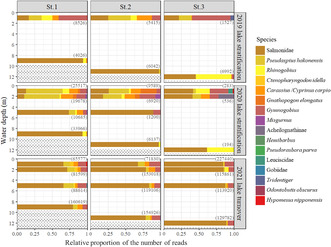

## 1 INTRODUCTION

Lakes are good sentinels for climate change, as they are generally sensitive to atmospheric temperature changes and are integrators of the surrounding landscape (Adrian et al., 2009; Schindler, 2009; Williamson et al., 2009). Indeed, recent climate change is anticipated to impact not only physicochemical factors in lake ecosystems such as water temperature and dissolved oxygen (DO) concentration but also biological factors such as distributions of organisms and community composition. In many temperate areas, lakes are thermally stratified with a surface warm layer (epilimnion), a bottom cold layer (hypolimnion), and a thermocline (temperature‐dependent density gradient) between these layers. Lakes are classified as dimictic when they have two stratification seasons, or as monomictic when they stratify only once per year. Climate change is predicted to affect lake mixing regimes greatly; some monomictic lakes will become permanently stratified systems and some dimictic lakes will become monomictic (Woolway & Merchant, 2019). Such a lack or reduction of vertical mixing will result in decreases in the oxygen supply from surface water to deep layers, which often leads to the formation of hypoxic or anoxic “dead zones” (Diaz & Rosenberg, 2008; North et al., 2014). These facts strongly suggest that climate change will narrow “survival zones” of lake ecosystems, especially for cold‐water species, by both increasing water temperatures from the surface and intensifying hypoxia from the lake bottom.

Cold‐water stenotherms like salmonids are expected to be particularly vulnerable to the survival‐zone shrinkage from climate change because they require cool and oxygenated water (Davis, 1975; Plumb & Blanchfield, 2009). In summer, for example, lake trout (*Salvelinus namaycush*) in a lake in northwestern Ontario, Canada, preferred a narrow range of microhabitats at depths of 6–15 m (characterized by water temperatures <15°C and DO > 4 mg L^−1^) due to expansion of the warm surface layer and hypoxia at the lake bottom (Plumb & Blanchfield, 2009). Moreover, increasing water temperatures and low DO have negative effects on early life stages of salmonids, such as by reducing hatching success, hypoxia tolerance, survival, and growth to the fry stage (Del Rio et al., 2019). Despite this evidence, few field studies have investigated the effects of both water temperature and DO on the detailed vertical distribution of cold‐water fishes during stratification (Plumb & Blanchfield, 2009; Rodrigues et al., 2022; Sellers et al., 1998). This is mainly due to the difficulty of tracking individual fish in aquatic systems. Researchers sometimes use echo‐sounder technology to determine fish distribution and biomass (Gonzalez & Gerlotto, 1998; Ohshimo, 2004). However, as echo sounders cannot clearly discriminate fish species, this method is better suited for aquatic environments where the subject fish species are predictable (Yamamoto et al., 2016). Moreover, in lakes with a diverse fish fauna, precise estimation of the distribution and biomass of individual species based on direct observation has been difficult if not impossible, especially along a vertical environmental gradient.

**FIGURE 1:**
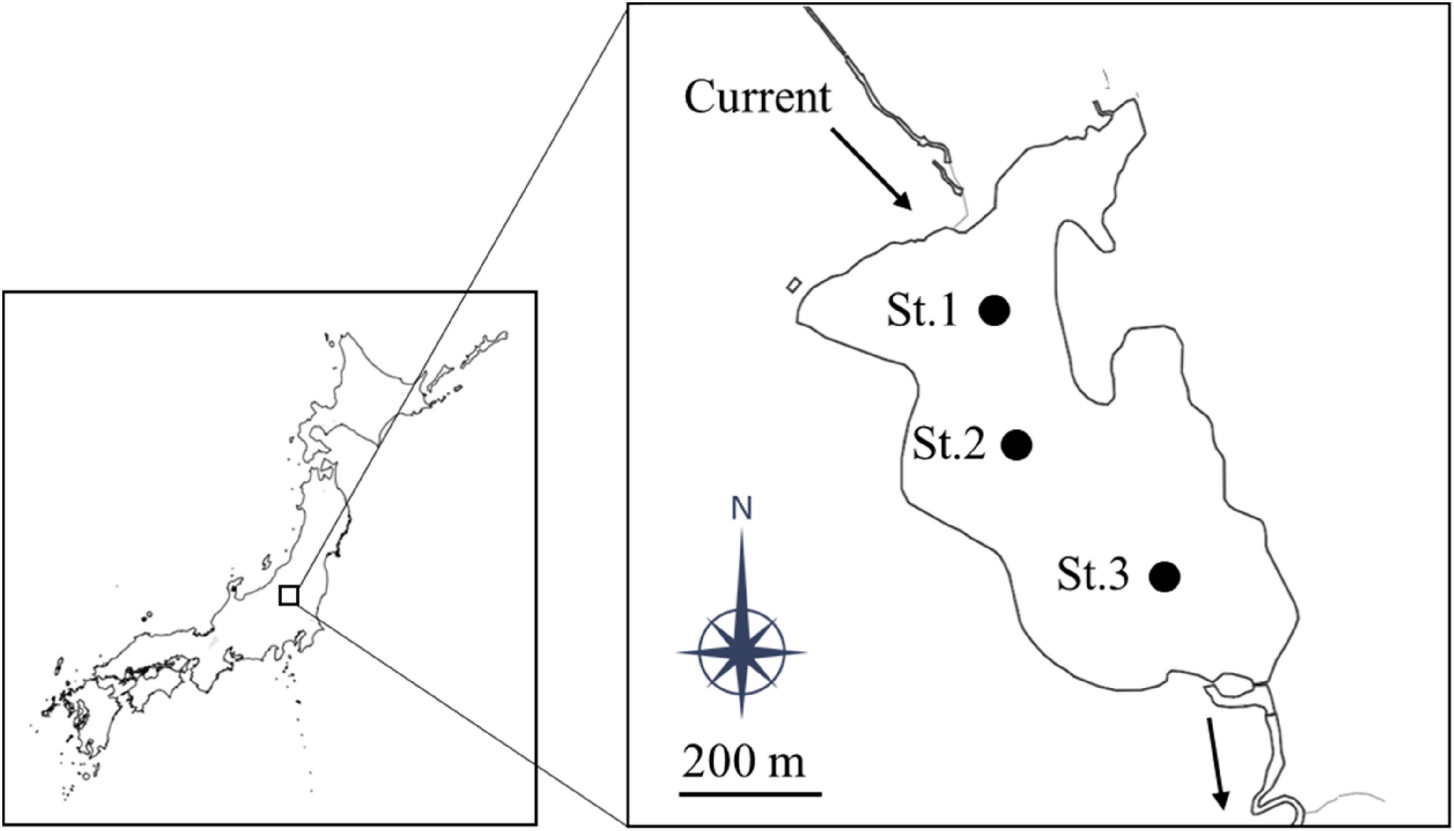
Map showing the location of Lake Yunoko, Tochigi prefecture, Japan, and the sampling stations. This map is based on the digital map (Basic Geospatial Information) published by Geospatial Information Authority of Japan.

Here, we examined how the abundance and diversity of lake fishes are vertically distributed in relation to environmental gradients by using environmental DNA (eDNA). First, we examined the vertical abundance (i.e., eDNA copy numbers) of three salmonid fishes, kokanee salmon (*Oncorhynchus nerka*), rainbow trout (*O. mykiss*), and masu salmon (*O. masou*) using quantitative PCR (qPCR). They are known as major top predators of freshwater ecosystems (Hossain et al., 2010; Negishi et al., 2019) and are quite common in the lake of interest, Lake Yunoko, Japan (Figure 1). These species have different oxythermal habitats (Tanaka et al., 1975; Mano, 1996; Matthews & Berg, 1997, see study species in Section 2: Materials and Methods). Therefore, we hypothesized that cold‐water species (i.e., kokanee salmon and masu salmon) are mainly distributed in deeper water layers during stratification, while species with high water temperature tolerance (i.e., rainbow trout) are distributed uniformly throughout the water column.

Next, we focused on the vertical distribution of diverse fish fauna using an eDNA metabarcoding. Our specific interest here was to better understand the overall characteristics of vertical eDNA distributions of fish communities in the lake during both stratification and turnover period. We then discuss the usefulness of the eDNA‐based distribution assessment for cold‐water fish species in the stratified lake, and the predictability of their habitat use and anticipated impacts of the narrowing survival zones in lakes under climate change.

## 2 MATERIALS AND METHODS

### 2.1 Study station and study species

Sampling was conducted in Lake Yunoko, Japan (Lat: 36.80049, Lon: 139.42444; altitude, 1478 m above sea level) on 30 August 2019, 6 August 2020, and 25 November 2021 (Figure 1). Lake Yunoko is a small (surface area, 0.35 km^2^; volume, 0.003 km^3^), eutrophic, and dimictic dammed lake in Nikko National Park (Murakami, 1991). The lake is covered with ice in winter. The maximum and average water depths are 14 and 7.4 m, respectively, and the average residence time of lake water is 25 to 30 days (Murakami, 1991).

Originally, Lake Yunoko was fishless until the early 1900s, but four salmonid species—kokanee salmon, masu salmon, rainbow trout, and brook trout (*Salvelinus fontinalis*)—have been introduced for recreational fishing. During spring to summer, 31,416 individuals (6895 kg) and 23,651 individuals (5130 kg) of rainbow trout over 20 cm in total length (TL) were stocked weekly into Lake Yunoko in 2019 and 2020, respectively, for recreational fishing by the National Federation of Inlandwater Fisheries Cooperatives. In addition, kokanee salmon at age 0+ (50,000 individuals in 2019 and 30,000 individuals in 2020) and masu salmon of the same age (10,000 individuals in both 2019 and 2020) were also stocked in the lake in June. Adults of rainbow trout are released into Lake Yunoko at two northern lakeshore points and one southern outflow point of Yukawa River, while kokanee salmon and masu salmon juveniles are released at a northern lakeshore point. Because the fish release date was different from that of our sampling, we believe that the vertical profiles of fish eDNA would not be affected by the fish releases.

These salmonid species have different oxythermal habitats: <13°C with DO >3 mg L^−1^ for kokanee salmon (Tanaka et al., 1975), <13°C for masu salmon (suitable DO for masu salmon was unavailable, Mano, 1996), and <19°C with DO > 3 mg L^−1^ for rainbow trout (Matthews & Berg, 1997).

### 2.2 Water sampling for eDNA

Lake water was sampled from the survey ship at various water depths using a Van Dorn water sampler. The Van Dorn water sampler was bleached with 10% commercial bleach (approx. 0.6% sodium hypochlorite) every time before collecting lake water at each water depth. Sampling depths were chosen with consideration for the thermocline (Figures S1 and S2). In 2019, water was sampled at depths of 1 and 9 m at St. 1, 1 and 11 m at St. 2, and 1 and 12 m at St. 3. In 2020, water was sampled at depths of 1, 2, 5, and 9 m at St. 1, 1, 2, 5, and 11 m at St. 2, and 1, 2, and 12 m at St. 3. In 2021, water was sampled at depths of 1, 2, 5, and 9 m at St. 1, 1, 2, 5, and 11 m at St. 2, and 1, 2, 5, and 12 m at St. 3. Near‐bottom water was collected from at least 50 cm above the lake bottom in order to avoid the collected water from containing sediments that could cause PCR inhibition, low DNA extraction rates, and/or contamination by old sedimentary eDNA. Sampled water (1 L) was poured into bottles that had been bleached with 10% commercial bleach and washed with Milli‐Q water. To prevent DNA degradation (Yamanaka et al., 2017), 1 mL of Osvan (Nihon Pharmaceutical, Tokyo, Japan, 10% m/w) was added to each bottle to achieve a final benzalkonium chloride concentration of 0.01%. The water bottles were placed on ice until the filtration.

### 2.3 Measurement of environmental factors

We measured the following environmental parameters using a handheld multiparameter meter (ProDSS; YSI, Yellow Springs, Ohio, USA) during water sampling at each station: water temperature (°C), water depth (m), pH of water, specific conductivity (μS cm^−1^), DO (mg L^−1^), and chlorophyll *a* concentration (mg L^−1^). Chlorophyll *a* in a water column at 10‐ to 12‐m depth in 2020 could not be obtained because of the length limitation of the handheld multiparameter meter (i.e., 10 m).

### 2.4 Water filtration and DNA collection

Filtration was performed on the day of water sampling. Each 1‐L water sample was filtered through a Sterivex filter unit (0.45 μm pore‐size; Merck Millipore, Darmstadt, Germany) using a 50‐mL syringe, and 1.8 mL RNAlater (Thermo Fisher Scientific, Waltham, MA, USA) was added for DNA preservation. As a blank control, we filtered 1 L Milli‐Q water from a bottle bleached with 0.1% sodium hypochlorite and washed with Milli‐Q water (i.e. bottle blank). In addition, 1 L Milli‐Q water poured into a bleached Van Dorn water sampler and was then collected in the same sterilized bottle and was filtered to check cross‐contamination from the Van Dorn water sampler (i.e. Van Dorn sampler blank).

Total eDNA was extracted from the Sterivex filter unit using a DNeasy Blood and Tissue Kit (Qiagen, Hilden, Germany) following Miya et al. (2016) and the Environmental DNA Sampling and Experiment Manual (Version 2.2, eDNA Society, 2020). The RNAlater in the Sterivex filter unit was removed by centrifugation at 2000× *g* for 3 min after gently shaking, and 440 μL of a mixture containing 220 μL of TE buffer, 200 μL of buffer AL, and 20 μL of proteinase K (Qiagen, Hilden, Germany) was added to the Sterivex filter unit. The filters were incubated on a rotary shaker at 20 rpm and 56°C for 60 min. The incubated solution was transferred into a new 2.0‐mL tube by centrifugation at 4000× *g* for 3 min, and eDNA was extracted by using the DNeasy Blood and Tissue Kit. We eluted the DNA in 100 μL AE buffer and stored at −20°C until analysis. To check for cross‐contamination during eDNA extraction, the same procedure was performed using nuclease‐free water, the buffers, and spin columns from the DNeasy Blood and Tissue Kit (i.e. extraction blank).

### 2.5 Primer and probe design for kokanee salmon and masu salmon

To detect the DNA of the three salmonid species, species‐specific real‐time PCR was conducted using the primer‐probe set presented by Minamoto et al. (2018) for rainbow trout (Table S1) and primer‐probe sets developed in this study for kokanee salmon and masu salmon. A primer‐probe set for sockeye salmon (the anadromous form of kokanee salmon) has already been designed using the mitochondrial cytochrome *c* oxidase subunit I (COI; Hellberg et al., 2010). However, in this study, we chose the *cytb* gene because the sequence information is available for many more salmonids than with the COI gene.

The accession numbers used for designing kokanee salmon and masu salmon primers are shown in Table S2. Sequences of *cytb* of the target species (kokanee salmon and masu salmon) and three sympatric salmonid species (rainbow trout, brook trout, and whitespotted char) were downloaded from the GenBank database (http://ncbi.nlm.nih.gov). We also downloaded the sequences of closely related salmonid species inhabiting nearby Lake Chuzenji: lake trout and brown trout. Nucleotide sequences were aligned using ClustalW algorithm (Thompson et al., 1994) with the default parameter settings of MEGA 5.2.2 software (Tamura et al., 2011). Sets of primers and TaqMan probes for kokanee salmon and masu salmon were designed using PrimerQuest Tool (Integrated DNA Technologies, Inc., Coralville, Iowa, USA). The designed primers and probes of kokanee salmon and masu salmon were as follows (Tables S3 and S4): kokanee salmon: O_nerka_CytB_F (5′‐GGATTAACTCCGATGCCGATAA‐3′), O_nerka_CytB_R (5′‐GGCTAAGGATGTTAGACCAAGAA‐3′), and O_nerka_CytB_P (5′‐FAM‐CCCTTACTTCTCATACAAAGACCTCCTGGG‐TAMRA‐3′); masu salmon: O_masou_CytB_F (5′‐CGACCACTGACCCAATTCTTAT‐3′), O_masou_CytB_R (5′‐GGCAATTTGGCCGATGATAATG‐3′), and O_masou_CytB_P (5′‐FAM‐AGGCATACCCGTAGAACACCCATT‐TAMRA‐3′). Because masu salmon contains some subspecies (i.e. *O. masou subsp*. in Lake Biwa and *O. masou masou* in northern Japan), species‐specific primers and probes were developed for a conserved region of the *cytb* gene in all subspecies of masu salmon (Table S4).

### 2.6 qPCR for three salmonid species

We assessed the vertical eDNA distribution for the three salmonid species using qPCR, which reflect the abundance and biomass of fish species (Baldigo et al., 2017; Lacoursière‐Roussel et al., 2016; Takahara et al., 2012) as well as associated with eDNA shedding (Rourke et al., 2022).

The eDNA samples were quantified by real‐time TaqMan qPCR using a LightCycler 480 Instrument (Roche Applied Science, Mannheim, Germany). Each Taqman reaction contained 900 nM of each primer (forward and reverse) and 125 nM of Taqman probe in a 1× PCR master mix (Taqman® Environmental Master Mix 2.0; Thermo Fisher Scientific), 2 μL of DNA template and nuclease‐free water. The total volume of each reaction mixture was 20 μL. The qPCR conditions were as follows: 95°C for 10 min, followed by 50 cycles of 95°C for 15 s and 60°C for 60 s. Six assays were performed for each sample.

A standard curve was developed by creating a six‐level standard curve dilution series (10 to 1*10^6^ copies μL^−1^). We assured that all data fell within the standard curve range of 10 to 1*10^6^ copies μL^−1^.

### 2.7 Testing the specificity of the designed assay

Genomic DNA was extracted from the gut tissues of two kokanee salmon individuals, one masu salmon individual, and two rainbow trout individuals using a DNeasy Blood & Tissue Kit (Qiagen) according to the manufacturer's protocol. Artificial DNA (526 bp of the *cytb* gene) of kokanee salmon, masu salmon, rainbow trout, brook trout, and whitespotted char were also designed to cover the primer and probe regions of salmonids with an extra 30 bases on each end (Thermo Fisher Scientific; GeneArt Strings DNA Fragments and Libraries) and used to test the specificity of the assay. The specificity of each assay was tested in triplicate using concentrations of 1.0 × 10^4^ to 1.0 × 10^7^ copies L^−1^ of target‐ and nontarget‐species DNA as a template (Table S5). The specificities of the primers and probes for kokanee salmon and masu salmon were first checked in silico using Primer‐BLAST (http://www.ncbi.nlm.nih.gov/tools/primer‐blast/), and then tested using artificial and gut‐tissue DNA of sympatric salmonid species (Table S5). We also checked the species‐specificity of the rainbow trout primer–probe set using kokanee salmon (*O. nerka*) and brook trout (*S. fontinalis*) inhabiting Lake Yunoko (Table S5).

### 2.8 Fish metabarcoding

We examined the vertical distribution of diverse fish fauna using an eDNA metabarcoding assay in which multiple species are identifiable from a single environmental water sample (Miya et al., 2015). The mitochondrial 12S rRNA of the extracted DNA was amplified using the fish‐universal primer pair MiFish‐U‐F (ACACTCTTTCCCTACACGACGCTCTTCCGATCTNNNNNNGTCGGTAAAACTCGTGCCAGC) and MiFish‐U‐R (GTGACTGGAGTTCAGACGTGTGCTCTTCCGATCTNNNNNNCATAGTGGGGTATCTAATCCCAGTTTG) primers with Illumina sequencing primer region and 6‐mer Ns (Miya et al., 2015). The total reaction volume was 12 μL containing 0.24 U KOD Fx Neo polymerase (Toyobo, Osaka, Japan), 1× PCR buffer for KOD Fx Neo, 0.4 mM dNTP mix, 0.3 μM of each MiFish primer, 2.0 μL template, and nuclease‐free water. The thermal cycle profile was 94°C for 2 min; 35 cycles of 98°C for 10 s, 61°C for 30 s, and 70°C for 30 s; and finally 70°C for 5 min. The first PCR was performed using eight replicates to mitigate false negatives (PCR dropouts). Thereafter, individual first PCR replicates were equally pooled. Ten microliters of each PCR product was purified using AMPure XP (Beckman Coulter, Brea, California, USA) and eluted with 40 μL of TE buffer. The purified first PCR products were used as templates for the second PCR.

The second PCR was performed to add MiSeq adaptor sequences and 8‐bp dual index sequences to both amplicon ends (Miya et al., 2015). The total reaction volume of the second PCR was also 12 μL containing 0.24 U KOD Fx Neo polymerase, 1× PCR buffer for KOD Fx Neo, 0.4 mM dNTP mix, 0.3 μM of each MiFish primer, 2.5 μL template, and nuclease‐free water. The thermal cycle profile was 94°C for 2 min; 10 cycles of 98°C for 10 s and 70°C for 30 s; and finally 68°C for 5 min. Equal volumes of second PCR products were mixed and isolated using E‐Gel SizeSelect II (Thermo Fisher Scientific, Waltham, MA, USA). Subsequently, the second PCR products were purified using a QIAquick Purification Kit (QIAGEN), and the concentration of the library was measured using a the 2100 bioanalyzer (Agilent Technologies) with the Agilent High Sensitivity DNA kit (Agilent Technologies) and diluted to 4 nM. After an alkaline denaturation, the library was sequenced in MiSeq (Illumina, San Diego, CA, USA) using the MiSeq Reagent Kit v2 300 cycles with 10% PhiX (Illumina). The metabarcoding dataset on 2021 were sequenced on a different run with the 2019–2020 dataset.

According to the environmental DNA sampling and experiment manual of the eDNA Society (Minamoto et al., 2021), salmonid fishes include difficult‐to‐identify species as the sequences for this group are similar. We therefore regarded the ASVs of all salmonid species as family Salmonidae. For a similar reason, we regarded two ASVs as family Leuciscidae, one ASV as family Gobiidae and one ASV as subfamily Acheilognathidae.

### 2.9 Bioinformatics

We generated Fastq files for index reads in MiSeq Reporter (Illumina). MiFish primer pairs were then removed from samples using cutadapt v2.8 (Martin, 2011) with default options. The ASV method was implemented using Divisive Amplicon Denoising Algorithm 2 (DADA2 v1.24.0; Callahan et al., 2016) in the R package with default settings. In DADA2, after quality filtering and trimming were performed, error rates were quantified using the learnErrors function, for the forward and reverse sequences independently. Sequences were then error corrected and merged to produce an ASV–sample matrix. After removal of ASVs with their lengths shorter than 135 or longer than 210 bases, chimeric ones were removed using the removeBimeraDenovo function in DADA2. Subsequently, contaminant ASVs were identified and removed using the R‐package decontam v1.16.0 (Davis et al., 2018). Finally, ASVs with less than 10 reads were removed as expected PCR errors. For the obtained ASVs, we assigned taxonomy through local BLAST searches using Claident version 0.9.2022.04.28 (Tanabe, 2021) (cldentseq with “animals_mt_species” for the local blast database, “qc” for the identification method, and default settings for the other options), which integrates BLAST+ (Camacho et al., 2009) and NCBI taxonomy‐based sequence identification tools (Huson et al., 2007). The taxonomic assignment of raw sequences was manually checked with the BLAST searches when the lowest identified taxa were at family‐ or genus level.

### 2.10 Statistics and reproducibility

We used a generalized linear mixed model (GLMM) based on a gamma distribution using the canonical log‐link function to examine the differences between the concentrations of kokanee salmon and rainbow trout eDNA at different depths, with station as a random effect. We performed principal component analysis (PCA) using the five environmental variables (water depth, water temperature, water pH, specific conductivity, and DO) collected during the 2019 and 2020 surveys. The environmental variables were standardized and centered. We used a GLMM based on a gamma distribution using the canonical log‐link function to examine the relationship between the concentrations of kokanee salmon and rainbow trout eDNA and PC1 and PC2 scores, with station as a random effect. Because the concentrations of masu salmon eDNA were below the detection limit (i.e., <10 copies μL^−1^) during stratification, we used a GLMM based on a binomial distribution to examine the relationship between eDNA detection (detectable or not detectable) and PC1 and PC2 scores with station as a random effect. We used the cbind function and a GLM with binomial distribution and the log‐link function to examine the differences between the proportion of the number of reads of fish species between depths. We applied the likelihood ratio test to compare five environmental variables which are deeper than 5 m depth between stations, with station as a random effect. All statistical analyses were conducted in R ver. 4.0.3 (R Development Core Team, 2020).

## 3 RESULTS

### 3.1 Environmental factors

Water temperature profiles at three stations showed that thermal stratification occurs during the summer in Lake Yunoko (Figures S1 and S2). The thermocline was confirmed between 0 and 2 m from the surface. DO in the 2019 survey was about 12 mg L^−1^ in the surface layer and decreased to 0 mg L^−1^ at 8–12‐m depth, which means that there was anoxia in the bottom layer. During the 2020 survey, spring water with high DO concentration was flowing into the lake near station St. 1, thus the water in the bottom layer there was not anoxic, as it was at St. 2 and St. 3 (Figure S2). The specific conductivity was 140–160 μS cm^−1^ in the surface layer and increased with depth. The pH of the surface layer was 7.5–8.1 and decreased with depth to a minimum of around 6.5. The chlorophyll *a* concentration in the 2019 survey was highest at 3–4‐m depth and lowest at 10–12‐m depth. In the 2020 survey, the chlorophyll *a* concentration was high at 2‐m depth and toward the bottom layer and lowest at 6–7‐m depth. In contrast, the environmental profiles during autumn turnover were relatively uniform throughout the water column (Figure S3).

### 3.2 eDNA distribution of salmonids

For the primer–probe sets of the three salmonid species, we confirmed that only the target species were detected from artificial and gut‐tissue DNA of sympatric salmonid species (Table S5). All negative controls including filtration, DNA extraction, and PCR blanks were negative in all the species‐specific qPCR (Table S5).

**FIGURE 2:**
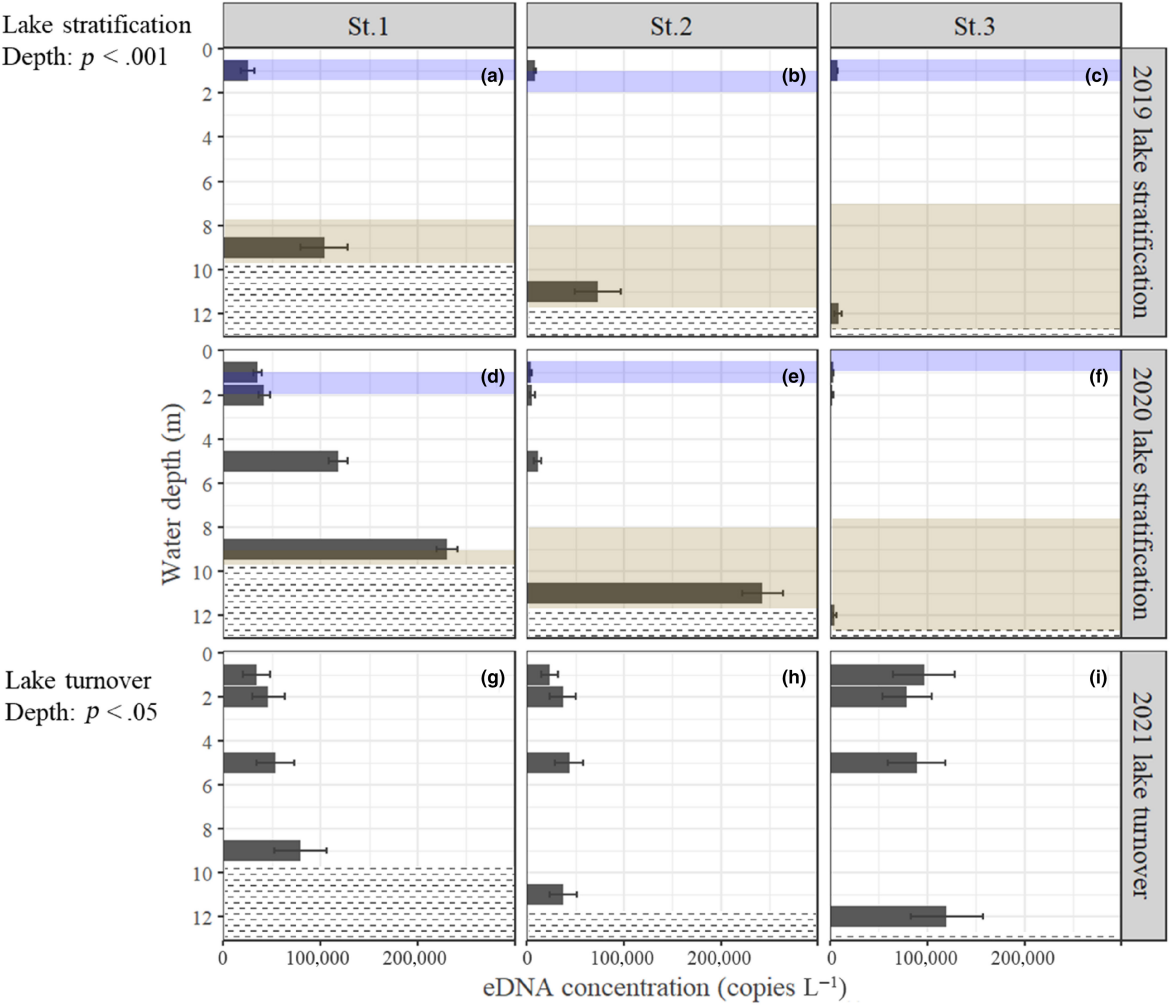
Vertical distribution of copy numbers (±SE) of kokanee salmon eDNA in the water column of Lake Yunoko. (a–c), 2019; (d–f), 2020; (g–i), 2021. Top line of dotted areas represents the location of the lake bottom. Note that thermocline depths are shaded in purple, and DO <3 mg L^−1^ are shaded in brown.

**FIGURE 3:**
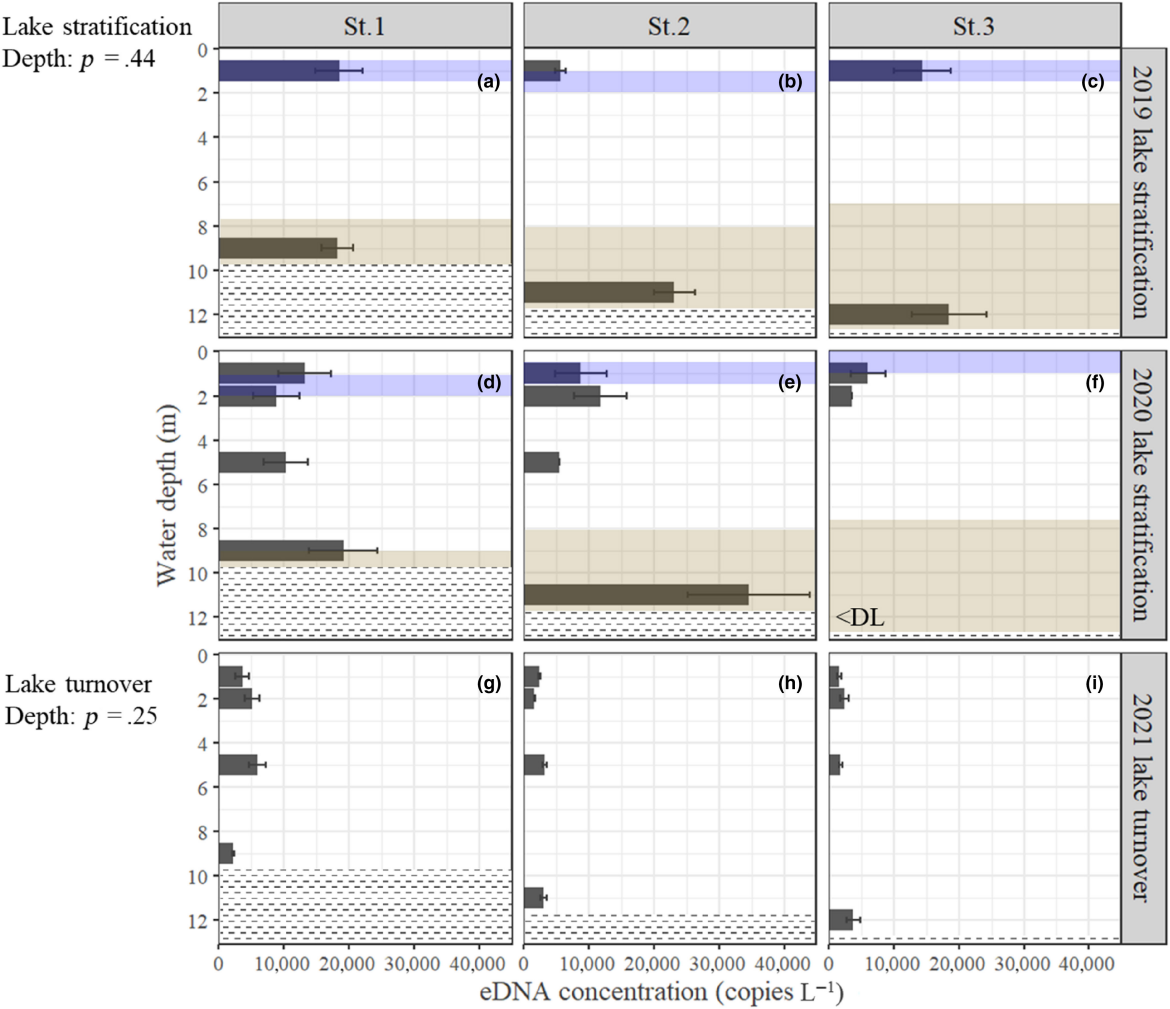
Vertical distribution of copy numbers (±SE) of rainbow trout eDNA in the water column of Lake Yunoko. (a–c), 2019; (d–f), 2020; (g–i), 2021. Note that the scale of x‐axis is different compared with Figure 2 because of low DNA copy number of this species. “DL” refers to the detection limit of 10 copies μL^−1^ (St. 3, 12‐m depth, 2020). Other conditions are the same as in Figure 2.

We detected eDNA of kokanee salmon and rainbow trout at all stations and depths (Tables S6 and S7). The eDNA of masu salmon was detected at all stations and depths except for two samples: St. 2 at 11‐m depth in the 2019 survey and St. 1 at 5‐m depth in the 2020 survey (Table S8). During lake stratification, the concentrations of kokanee salmon eDNA were higher in the bottom layer than in the surface layer except at St. 3, where the eDNA concentration was low throughout the water column (Figure 2). In the 2020 survey, the concentration of kokanee salmon eDNA at St. 2, 11‐m depth (243,000 ± 52,250 copies L^−1^) was 60 times that at 1 m (4020 ± 1452 copies L^−1^). There were significant differences between depths in the concentrations of kokanee salmon eDNA (GLMM, Estimate = 0.21, *p* < .001). During autumn turnover, the concentrations of kokanee salmon eDNA in the deepest layers in St. 1 and St. 3 were slightly higher than surface and medium layers, and there were significant differences among depths (GLMM, Estimate = 0.04, *p* < .05; Figure 2). In contrast, the concentrations of rainbow trout eDNA were lower than those of kokanee salmon eDNA and did not significantly differ between depths during both stratification and turnover periods (GLMM, lake stratification; *p* = .44; lake turnover; *p* = .25; Figure 3). The concentrations of masu salmon eDNA were below the detection limit (i.e., <10 copies μL^−1^) at all stations and depths and could not be quantified during stratification. The eDNA concentrations of masu salmon during autumn turnover did not significantly change between water depths (GLMM, *p* = .11; Figure S4).

### 3.3 Relationship between eDNA concentrations and environmental variables

We applied PCA to assess the importance of five environmental variables that might affect the vertical distribution of kokanee salmon, masu salmon, and rainbow trout. The first and second principal components contributed 87.8% and 9.3%, respectively, and the cumulative contribution of these two components reaches 97.1% (Table 1). PC1 was strongly and positively correlated with water depth and was also strongly and negatively correlated with water temperature, DO, and pH of water (Table 1, Figure S5), indicating that high PC1 values correspond to the deeper environments with low water temperature, low DO, and low pH of water (Figures S1 and S2). PC2 was not strongly correlated with water depth, suggesting that this axis does not reflect the surface or deep‐layer environment.

**TABLE 1:** Correlation matrix of principal components 1 and 2 (PC1, PC2) from principal components analysis (PCA) of environmental variables in the water column during lake stratification at Lake Yunoko, Japan.

Variables	PC1 (87.8%)	PC2 (9.3%)
Water temperature	**−0.43**	**0.53**
Water depth	**0.46**	−0.28
Specific conductivity	0.40	**0.80**
Dissolved oxygen	**−0.47**	−0.05
pH of water	**−0.47**	−0.04

*Note*: The environmental variables were standardized and centered.

Values >0.40 are listed in boldface and indicate strong loadings on each principal component (Nicholson & Clements, 2020).

**FIGURE 4:**
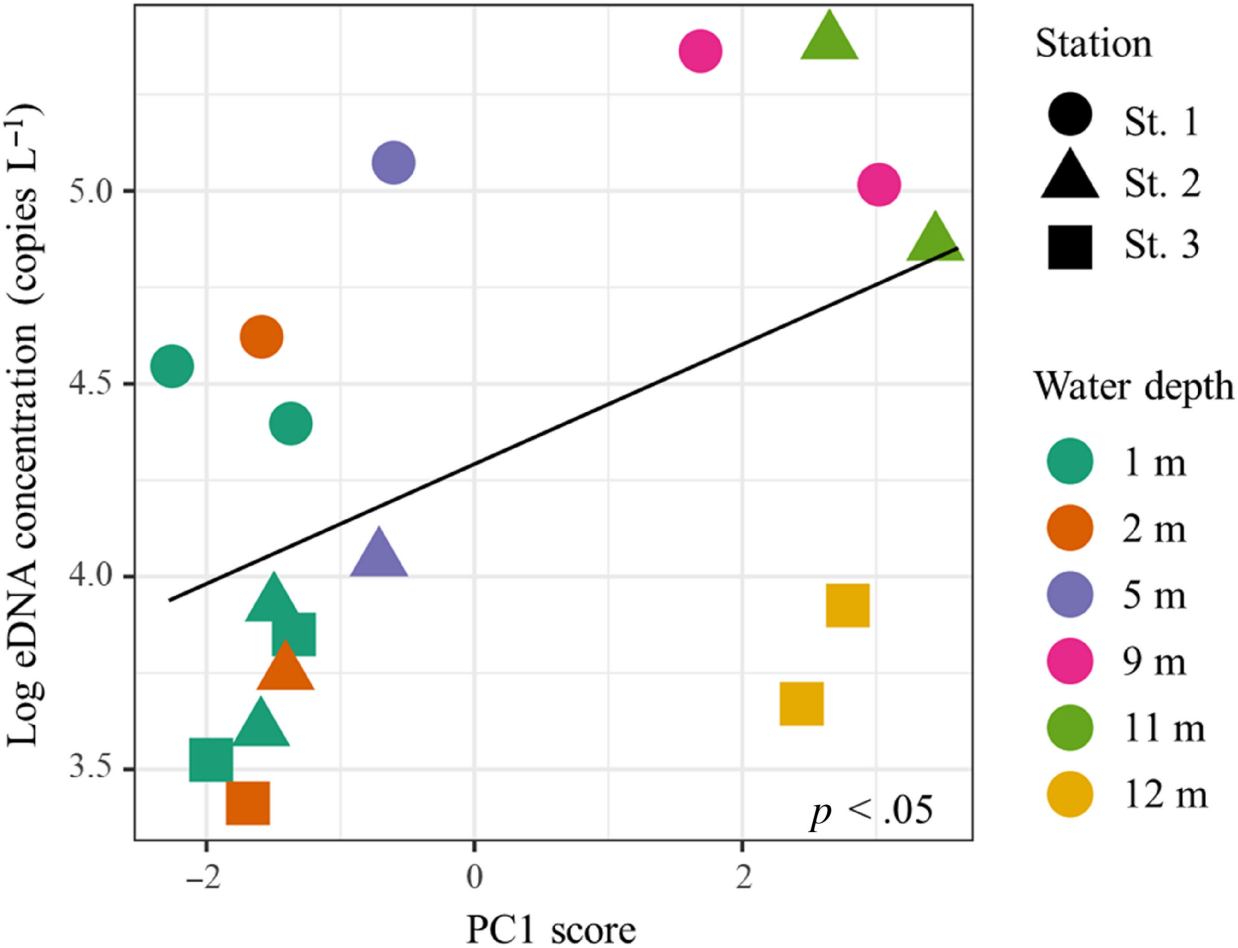
Relationship between the PCA score (PC1) and the log_10_ of copy number of kokanee salmon eDNA in Lake Yunoko. The linear regression equation model is *y* = 0.1534*x* + 4.2926 and *R* ^2^ value of .24.

The eDNA concentrations of kokanee salmon during lake stratification were positively correlated with PC1 and not with PC2 (Table 2). PC1 describes the deeper environments, so the eDNA of this species was mainly distributed in the bottom layer. On the other hand, the eDNA concentrations of rainbow trout and the detection rate for masu salmon eDNA were not associated with PC1 or PC2 (Table 2, Table S9), suggesting that the eDNA distribution of these species did not correlate with any environmental variables. The eDNA concentrations of kokanee salmon were positively correlated with the PC1 scores (*p* < .05; Figure 4). In contrast, the PC1 scores of rainbow trout were not correlated with their eDNA concentrations (*p* = .61). These indicate that eDNA of kokanee salmon was detected more generally in the deepest layers of the lakes, while that of rainbow trout was detected uniformly throughout the water column.

**TABLE 2:** Results of the GLMM for the relationship between log_10_ of the copy number of kokanee salmon and rainbow trout eDNA and the first and second PCA factors (PC1, PC2) during lake stratification.

Common name	Variables	Estimate	SE	*p* value
Kokanee salmon	(Intercept)	10.61	0.26	
	PC1	0.41	0.14	**<.01**
	PC2	−0.08	0.37	.83
Rainbow trout	(Intercept)	9.38	0.40	
	PC1	0.16	0.20	.44
	PC2	0.44	0.71	.53

Bold value represents significance at *p* < .05.

We compared five environmental variables which are deeper than 5 m depth (i.e., eDNA concentrations of kokanee salmon in these depth ranges were different between stations) between stations to examine why kokanee salmon eDNA were not distributed between medium and deep depth layer in St. 3. DO concentration, pH, and Chl‐a concentration were significantly different between all stations (*p* < .05, Figure S6). Water temperature and specific conductivity in St. 3 were different compared with those in St. 1 and St. 2 (*p* < .05). Overall, water temperature, DO concentration, and conductivity were low in St. 3 compared with St. 1 and St. 2.

### 3.4 Fish metabarcoding

From the raw dataset, we generated 551 amplicon sequence variants (ASVs; 1,653,824 reads). Of these, 217 ASVs (1,629,556 reads) were assigned to a fish species or genus in the taxonomy database, and 48 ASVs (9451 reads) were assigned to other taxonomic levels (e.g. Class: Mammalia and Aves). Seven ASVs were removed from the analysis because of food‐fish species and species which is not native to Japan. For the remaining 279 ASVs, no taxonomic name was assigned.

Based on taxonomic identification, 35 out of 217 fish ASVs were classified to the species level: *Pseudaspius hakonensis*, 19 ASVs; *Ctenopharyngodon idella*, 5 ASVs; *Gnathopogon elongatus*, 6 ASVs; *Odontobutis obscurus*, 1 ASV; *Hypomesus nipponensis*, 2 ASVs; and *Pseudorasbora parva*, 2 ASVs. Despite this, 111 out of 217 ASVs were assigned to a species or genus of salmonid fishes by the BLAST searches. Of those not identified to the species level, 39 ASVs were identified to the genus level: *Gymnogobius*, 28 ASVs, *Rhinogobius*, 4 ASVs, *Misgurnus*, 4 ASVs, *Hemibarbus*, 2 ASVs, and *Tridentiger*, 1 ASV. Because of the genus *Carassius* and *Cyprinus carpio* are difficult to identify, we regarded 28 ASVs of these species as *Carassius/Cyprinus carpio*.

**FIGURE 5:**
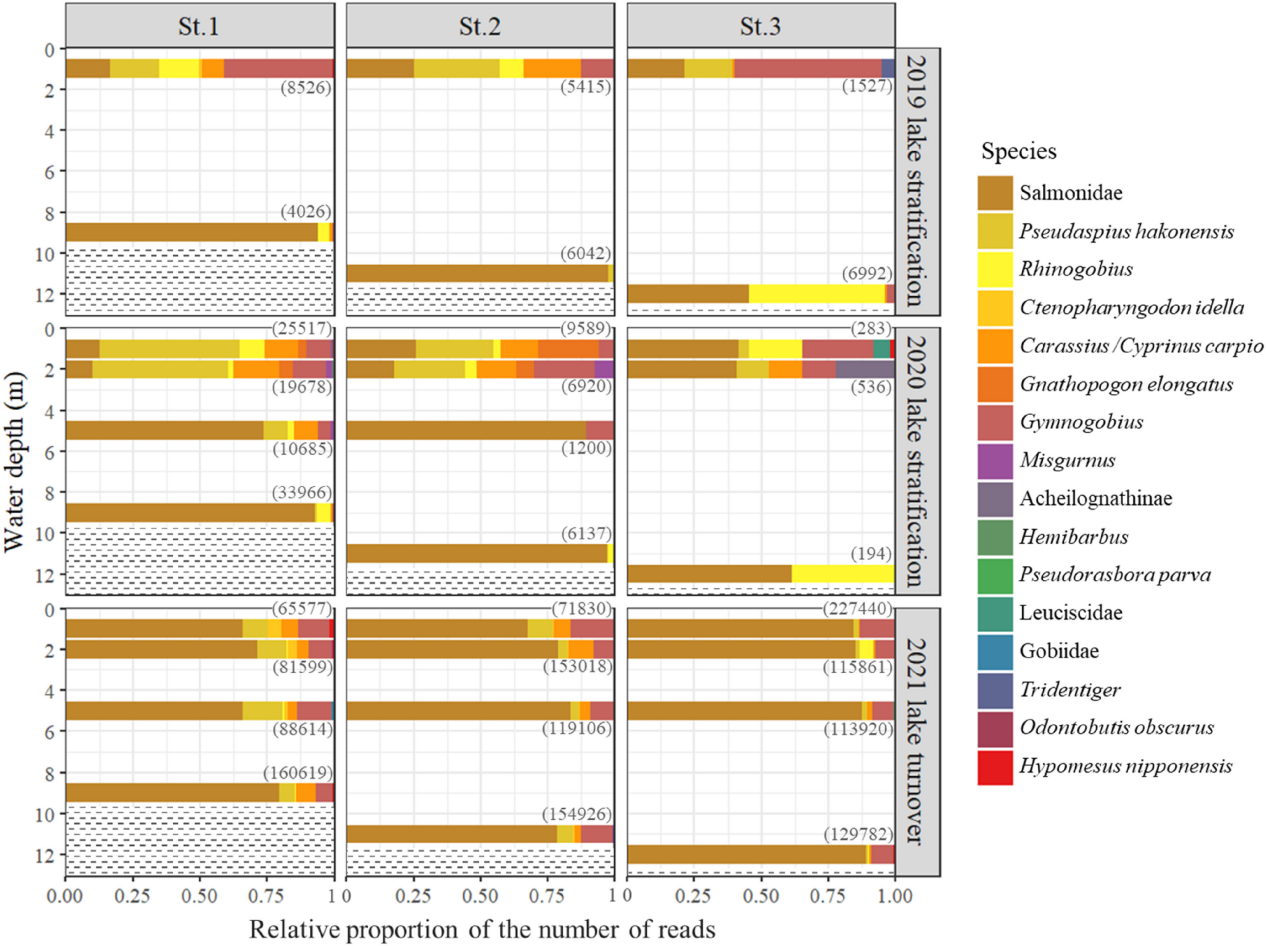
Relative proportion of fish species by depth at three stations in Lake Yunoko, Japan, as detected by the number of reads (i.e., the number of reads per taxon/total number of reads at each depth) during both stratification and turnover periods. The total numbers of reads at each depth are shown in parentheses. Top line of dotted areas represents the location of the lake bottom.

Numbers of taxa (i.e., total numbers of species, genera, and families) were higher at the shallowest depths (7.0 ± 2.0 at 1‐m depth at all stations) than at the deepest depths (4.2 ± 1.7 at 9–12‐m depth at all stations), and it differed significantly between depths (GLMM, Estimate = −0.05, *p* < .01, Figure 5) during stratification. Salmonidae accounted for most of the number of reads at all stations during the three‐year survey, and there were significant differences in the proportion of the number of reads between depths (GLM, Estimate = 0.17, *p* < .001). *Pseudaspius hakonensis*, *Gnathopogon elongatus*, *Carassius/Cyprinus carpio*, and genus *Gymnogobius* were mostly detected above the thermocline, and there were significant differences in the proportion of the number of reads between depths (GLM, *P. hakonensis*: Estimate = −0.41, *p* < .001; *Gnathopogon elongatus*: Estimate = −0.60, *p* < .001; *Carassius/Cyprinus carpio*: Estimate = −0.31, *p* < .001; genus *Gymnogobius*: Estimate = −0.32, *p* < .001). These results showed that the relative proportions of the number of reads of each species changed at different depths in the lake during stratification. Fish assemblages during autumn turnover were relatively uniform throughout the water column (Figure 5).

## 4 DISCUSSION

### 4.1 Impacts of warming and hypoxia on the distribution of cold‐water fishes

Recent climate change appears to be affecting various lake environments, such as by extending the period of thermal stratification and promoting the spread of hypoxia (Jenny et al., 2016; Winder & Schindler, 2004). Increases in surface water temperature could extend the duration of thermal stratification, which limits the oxygen supply from surface water to the lake bottom (North et al., 2014; Woolway et al., 2021). In addition, warm surface water increases phytoplankton production and cell biomass sinking to the deeper layers, resulting in an increase in bacterial oxygen consumption and the spread of hypoxia in lake bottom environments. In our study lake, there was stratification during summer. DO concentrations were above 10 mg L^−1^ in warm surface water but decreased to 0 mg L^−1^ in the cold bottom water, showing the development of anoxia at the greatest depths.

During stratification, habitat use by cold‐water stenotherms like salmonids is greatly limited because of the lack of areas with the preferred water temperature and adequate DO (Plumb & Blanchfield, 2009). In this study, we used specific primer–probe sets for three salmonid species, and the eDNA concentrations of kokanee salmon were higher in the bottom layer than in the surface and middle layers. In addition, we found that the eDNA concentrations for this species were correlated with the low water temperature, low DO, and low pH in the lake bottom environments. These findings may indicate that cold‐water species were mainly distributed at deeper cold‐water layers, avoiding warm surface‐water. Considering the habitat use of surface and medium depth waters, our results showed that rainbow trout eDNA was distributed uniformly throughout the water column compared with kokanee salmon eDNA during stratification. Suitable oxythermal habitats for kokanee salmon and rainbow trout are <13°C with DO >3 mg L^−1^ and <19°C with DO >3 mg L^−1^, respectively (Matthews & Berg, 1997; Tanaka et al., 1975). Thus, rainbow trout may have high water‐temperature tolerance compared with kokanee salmon. The concentrations of masu salmon eDNA were below the detection limit (i.e., <10 copies μL^−1^) at all stations during stratification, which may partly because their low stocking biomass (see Section 2: Materials and Methods).

Our results showed that rainbow trout eDNA were distributed all layers while kokanee salmon eDNA were distributed between medium and deep depth layer. Given that the above preferred habitats of kokanee salmon and rainbow trout, they could have been predominantly distributed between depths of 4–8 m and 1–8 m in our study lake (Figures 2 and 3, Figure S7). These suggest that kokanee salmon preferred deep, cool waters and that low water temperature may be more of a constraint on their habitat use than high DO. Also, chum salmon (*O. keta*) is known to stay in deep, cold layers during anadromous migrations to minimize metabolic energy costs (Tanaka et al., 2000). Our eDNA results suggest that kokanee salmon may minimize their metabolic energy costs in deep, cold waters during summer. These observations suggest that cold temperature may be a primary factor determining the vertical distribution of kokanee salmon in Lake Yunoko.

However, because the deep, cold layers are under anoxic or hypoxic conditions, those areas may be physiologically stressful for salmonids. We found that during stratification, kokanee salmon eDNA were not distributed in St. 3 where water temperature, DO concentration, and conductivity between depths of 5–8 m tended to be slightly low compared with St. 1 and St. 2 (Figure 2, Figure S6). Considering the preferred habitats for kokanee salmon (see above), they may avoid regions of low DO environment. If climate warming increases hypoxia, the survival zone of kokanee salmon could be reduced to a critical depth interval, resulting in a reduction of their populations. Besides, the recent spread of hypoxia due to climate change is anticipated to be amplified by excessive nutrient inputs such as agricultural fertilizer runoff and sewage discharge (Breitburg et al., 2018). Therefore, the impact of warm temperatures and low DO on the habitat use of cold‐water fishes may be accelerated in eutrophic lakes. Notably, despite general trends, the narrowing of survival zones is likely to be spatially heterogeneous and dependent on environmental conditions such as lake topography and the proportion of spring‐water inflow. Evaluating the interactive effects of large‐scale environmental changes such as climate warming and eutrophication and local lake characteristics on overall habitat volumes of cold‐water fishes is a promising direction for future research.

### 4.2 Vertical eDNA distributions of other fishes and factors affecting eDNA concentrations

Our results from other fish species also suggest that the numbers of taxa as detected by eDNA changed at different depths in the lake. Salmonid fishes comprised most of the number of reads at all stations, probably because four salmonid species have been stocked in this lake, resulting in high densities and eDNA concentrations across the water column. The most dramatic differences in community composition were seen in samples from above and below the thermocline: some species (e.g., *P. hakonensis* and *G. elongatus*) were detected only at the shallower depths in the water column, resulting in higher species diversity above the thermocline than below. Another study has also suggested that cold‐water fish eDNA could only be detected in the deepest layers of lakes, whereas warm‐water fish eDNA was much more abundant above the thermocline during summer stratification (Littlefair et al., 2021). Although fish distribution patterns can change depending on their developmental stage and reproductive period, our results show that fish eDNA was distributed across the water column in Lake Yunoko during stratification in a species‐specific way. Furthermore, this suggests that climate change will cause a large disturbance to the vertical distribution of lake fish species and thus affect their populations and interactions.

Another possible explanation for the distribution of fish eDNA at different depths during summer stratification is water‐temperature‐dependent degradation. In Ayu sweetfish (*Plecoglossus altivelis altivelis*), the half‐decay times of time‐dependent eDNA degradation are 19.55 h at 10°C and 4.89 h at 20°C (Tsuji et al., 2017). This means that the degradation rate of Ayu sweetfish eDNA at 20°C is about 4 times that at 10°C. In our lake, average water temperatures at the shallowest and deepest depths were 17.4°C and 10.2°C, respectively, and thus the eDNA concentrations at shallower depths might be underestimated. Moreover, eDNA is reportedly more concentrated in aquatic sediments than in surface water (Barnes & Turner, 2016; Turner et al., 2015). In areas with a high density of salmonids, their feces rapidly sink and accumulate on the sediments (Reid et al., 2009; Turner et al., 2015). Thus, the reason for high concentrations of kokanee salmon eDNA at the deepest depths might be that eDNA particles sink and/or old sedimentary eDNA has been resuspended from the lake bottom. It is necessary that future studies using echo sounders together with the more detailed vertical eDNA sampling would uncover the whole picture of accurate habitat use of salmonids in the deep waters.

### 4.3 Future prospects

In this study, we were able to describe the overall characteristics of the vertical distribution of fish communities in a lake ecosystem using eDNA methods. During summer stratification, the narrow habitat use of cold‐water top predators may affect the distribution and behavior of other organisms in lake food webs. In boreal lakes in Canada, for example, cold‐water lake trout retreat from warmer nearshore habitats to cooler offshore habitats and did not much depend on nearshore food resources (Bartley et al., 2019). This behavioral response of a top predator to the warming of littoral habitat reduces top‐down effects in nearshore foraging habitats as the species comes to depend more heavily on deep offshore habitats, thereby altering carbon flow throughout the entire lake ecosystem. Our next challenge is to quantify any changes in abundance or biomass of top predators that may indirectly impact the abundance of other fish species under climate warming scenarios. It is essential to keep monitoring the distribution of cold‐water species in various types of lakes, and eDNA could provide information for narrowing the critical survival zones of fish populations under climate change.

## AUTHOR CONTRIBUTIONS

**Kayoko Fukumori:** Conceptualization (lead); data curation (lead); formal analysis (lead); funding acquisition (equal); investigation (lead); methodology (lead); project administration (lead); resources (equal); software (equal); supervision (equal); validation (equal); visualization (equal); writing – original draft (lead); writing – review and editing (equal). **Natsuko I. Kondo:** Conceptualization (lead); data curation (equal); formal analysis (equal); funding acquisition (lead); investigation (lead); methodology (lead); project administration (equal); resources (lead); software (equal); supervision (equal); validation (equal); visualization (lead); writing – original draft (equal); writing – review and editing (equal). **Ayato Kohzu:** Conceptualization (equal); data curation (equal); formal analysis (equal); funding acquisition (equal); investigation (lead); methodology (equal); project administration (equal); resources (equal); software (equal); supervision (equal); validation (equal); visualization (equal); writing – original draft (equal); writing – review and editing (equal). **Kenji Tsuchiya:** Conceptualization (equal); data curation (equal); formal analysis (equal); funding acquisition (equal); investigation (lead); methodology (equal); project administration (equal); resources (equal); software (equal); supervision (equal); validation (equal); visualization (equal); writing – original draft (equal); writing – review and editing (equal). **Hiroshi Ito:** Conceptualization (equal); data curation (lead); formal analysis (lead); funding acquisition (equal); investigation (equal); methodology (equal); project administration (equal); resources (equal); software (lead); supervision (equal); validation (equal); visualization (equal); writing – original draft (equal); writing – review and editing (equal). **Taku Kadoya:** Conceptualization (lead); data curation (equal); formal analysis (equal); funding acquisition (lead); investigation (lead); methodology (equal); project administration (lead); resources (equal); software (equal); supervision (lead); validation (equal); visualization (equal); writing – original draft (equal); writing – review and editing (lead).

## CONFLICT OF INTEREST STATEMENT

The authors declare no conflicts of interest.

## DECLARATIONS

The discarded gut tissues of salmonids, which were originally processed for human consumption by a recreational fisherman in Lake Yunoko, were used to check the species‐specificity of the primer‐probe sets of three salmonid species. No fish were sacrificed or injured in the present study. For these reasons, no ethical approval is required in this case.

## Supporting information


Data S1.

## Data Availability

The raw data from this study are available through NCBI's Sequence Read Archive (DRA accession number for each sample: DRX360206–DRX360222 and DRX423488–DRX423499).
