# Supplementary material for: Vertical eDNA distribution of cold‐water fishes in response to environmental variables in stratified lake
Source: Ecol Evol. 2024 Mar 18;14(3):e11091. doi: 10.1002/ece3.11091 (PMC10945234; doi:10.1002/ece3.11091)
Supplement: Supplementary file 1 — Data S1. [file ECE3-14-e11091-s001.docx]

**Supporting information**

**Vertical eDNA distribution of cold-water fishes in response to environmental variables in stratified lake**

Kayoko Fukumori^1*^, Natsuko I Kondo^1^, Ayato Kohzu^2^, Kenji Tsuchiya^2^, Hiroshi Ito^1^, Taku Kadoya^1^

^1^Biodiversity Division, National Institute for Environmental Studies (NIES), 16-2, Onogawa, Tsukuba, Ibaraki, 305-8506, Japan

^2^Regional Environment Conservation Division, National Institute for Environmental Studies (NIES), 16-2, Onogawa, Tsukuba, Ibaraki, 305-8506, Japan

*Address correspondence to Kayoko Fukumori, sandgoby2000@yahoo.co.jp

Table S1. PCR primer–probe set for rainbow trout (Minamoto et al. 2018)^a^ and consensus sequence information for rainbow trout and four sympatric salmonid species. Footnotes show the differences in the number of base pairs from the representative sequence for each species.


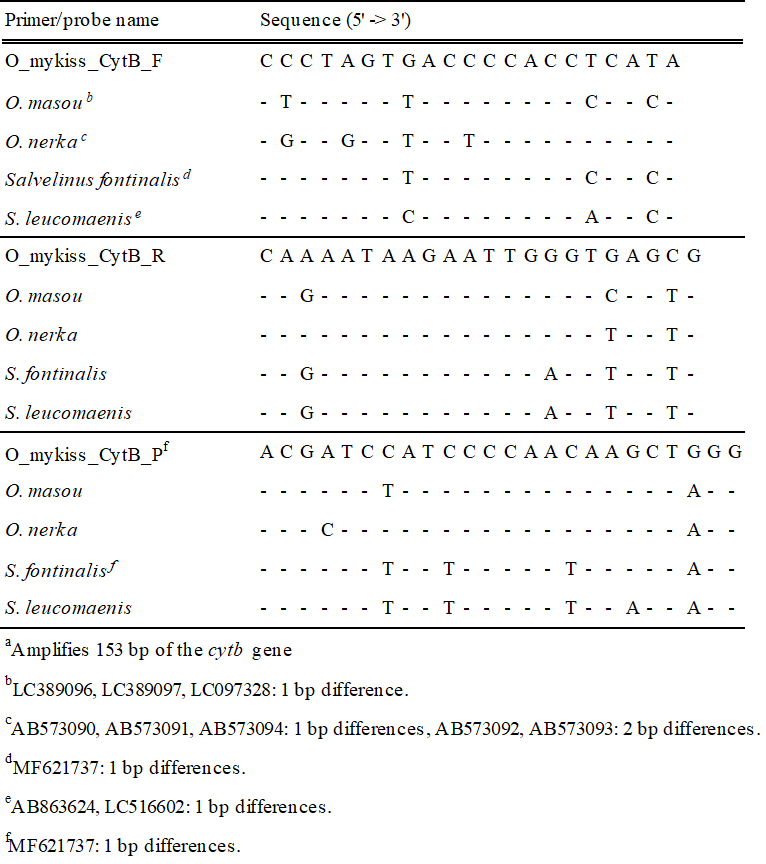


Table S2. Accession numbers for the nucleotide sequences used for designing kokanee salmon and masu salmon PCR primer–probe sets.


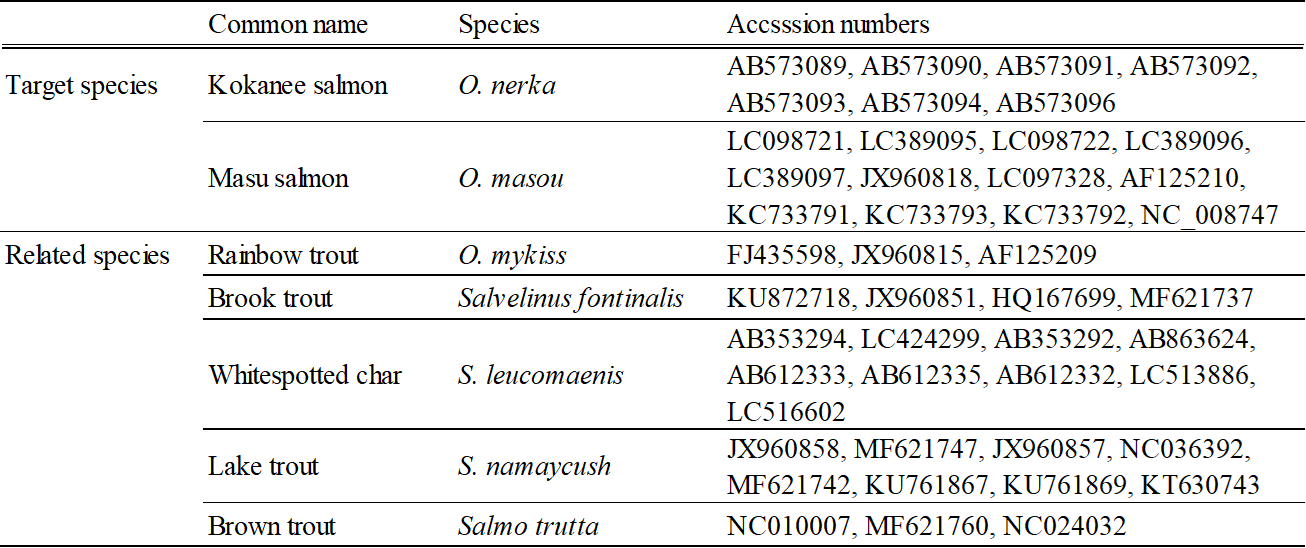


Table S3. PCR primer–probe set for kokanee salmon^a^ and consensus sequence information for kokanee salmon and four sympatric salmonid species. Footnotes show the differences in the number of base pairs from the representative sequence for each species.


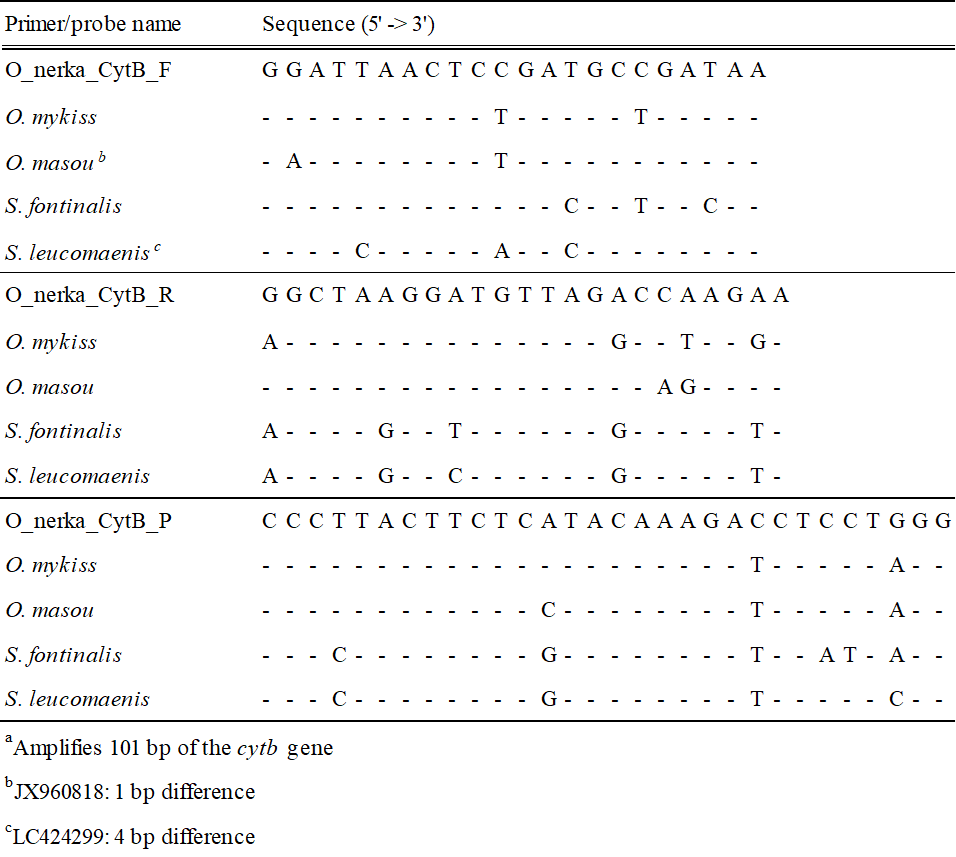


Table S4. PCR primer­–probe set for masu salmon^a^ and consensus sequence information for masu salmon and four sympatric salmonid species. Footnotes show difference of the number of base pairs from the representative sequence of each species.


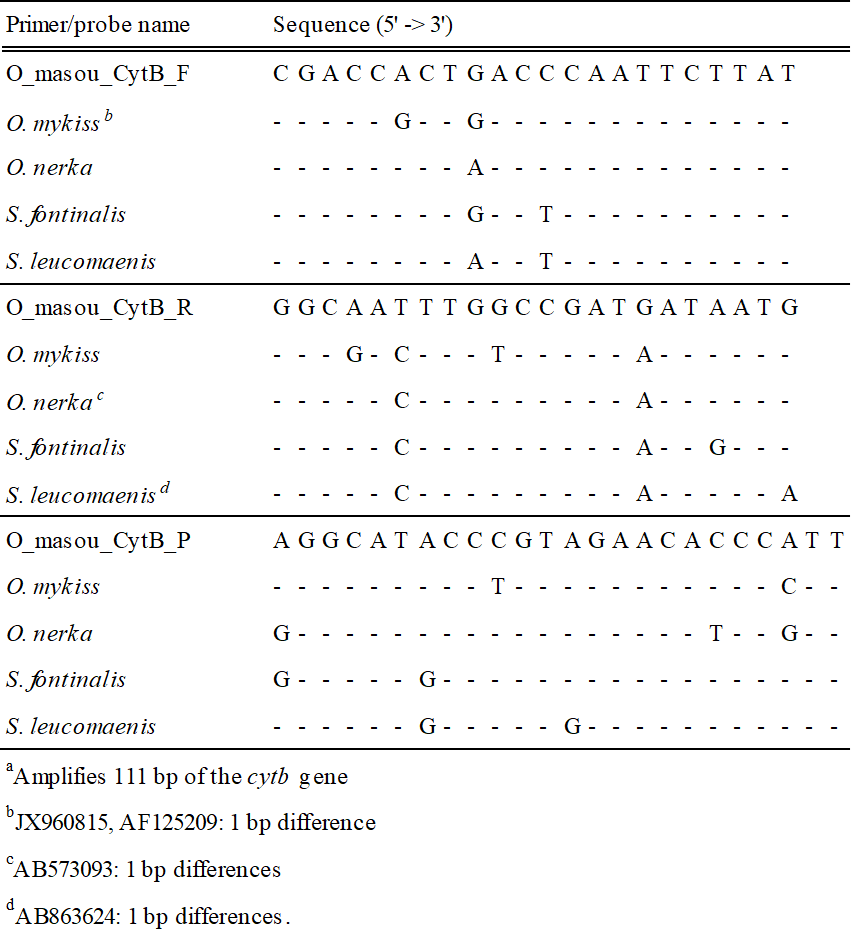


Table S5. Results of qPCR using DNA from target and non-target species as part of the process to develop a new assay system.


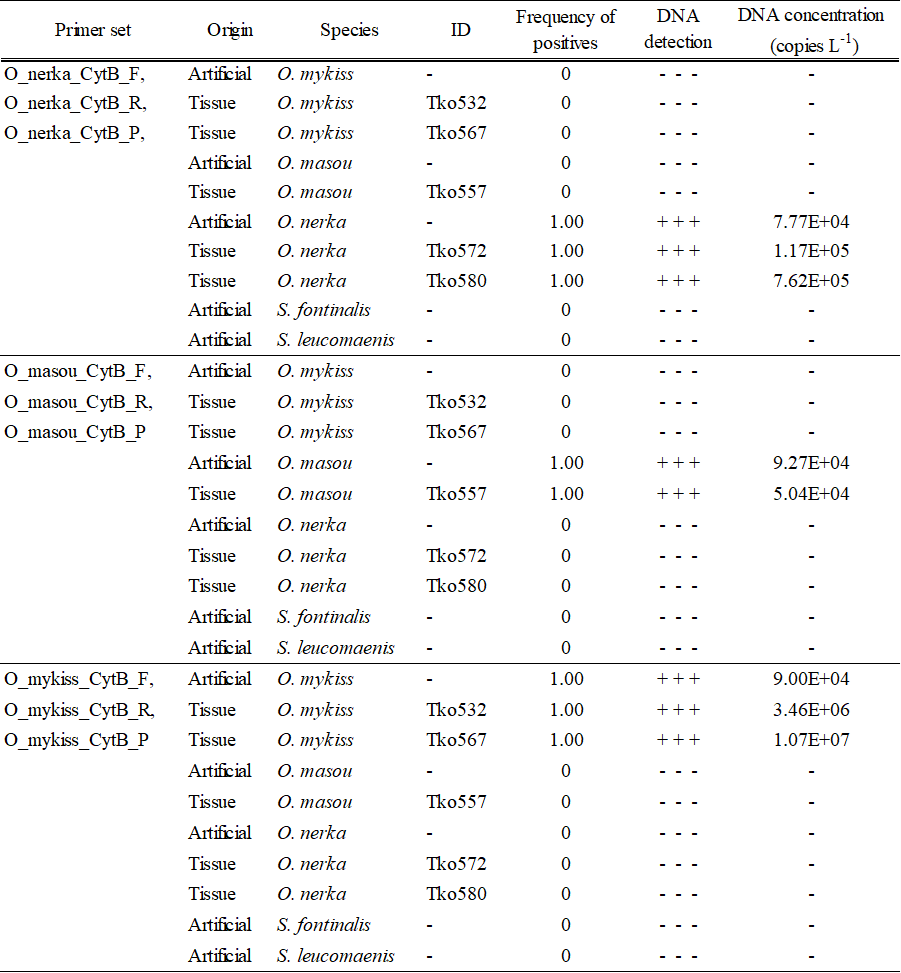


The negative controls were not amplified or the values were out of standard curve ranges.

Table S6. Results of positive (+) and negative (-) eDNA detection per six PCR replicates for kokanee salmon*.* ND, no data.


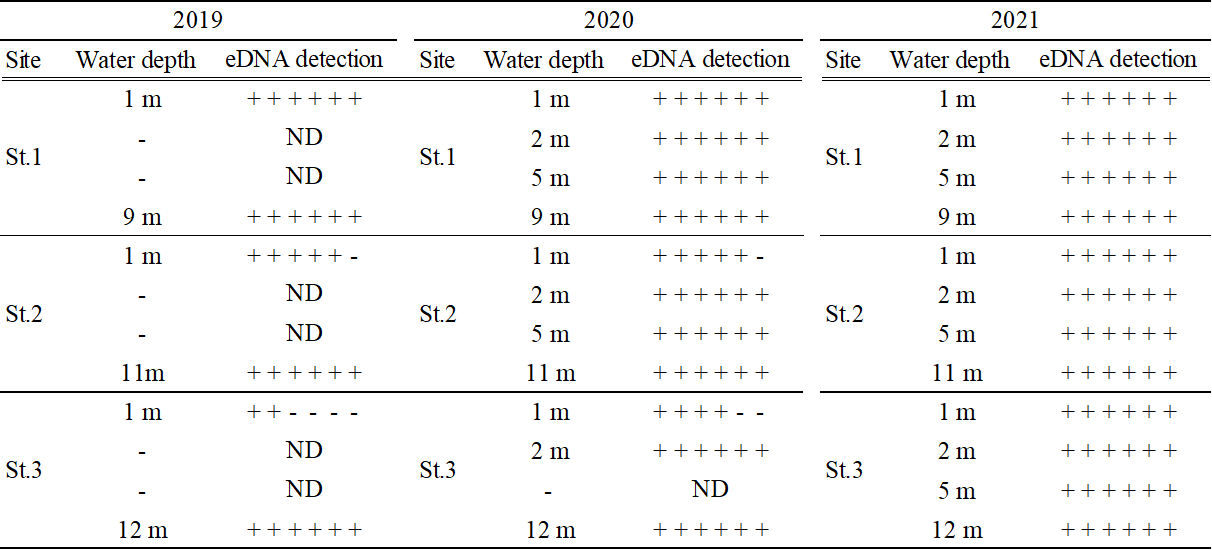


Table S7. Results of positive (+) and negative (-) eDNA detection per six PCR replicates for rainbow trout. ND, no data.


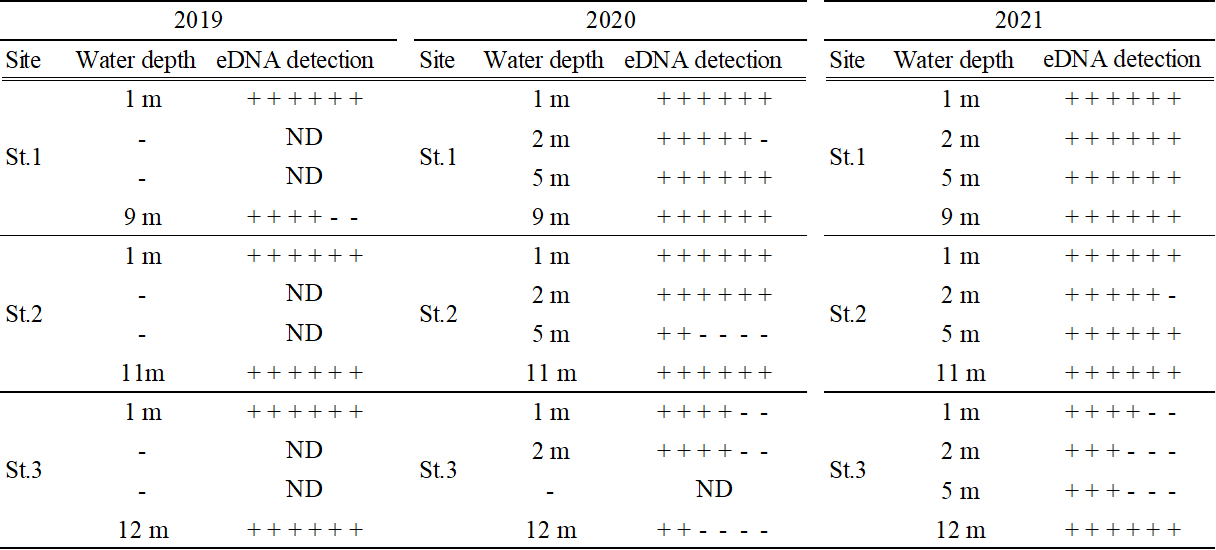


Table S8. Results of positive (+) and negative (-) eDNA detection per six PCR replicates for masu salmon*.* ND, no data.


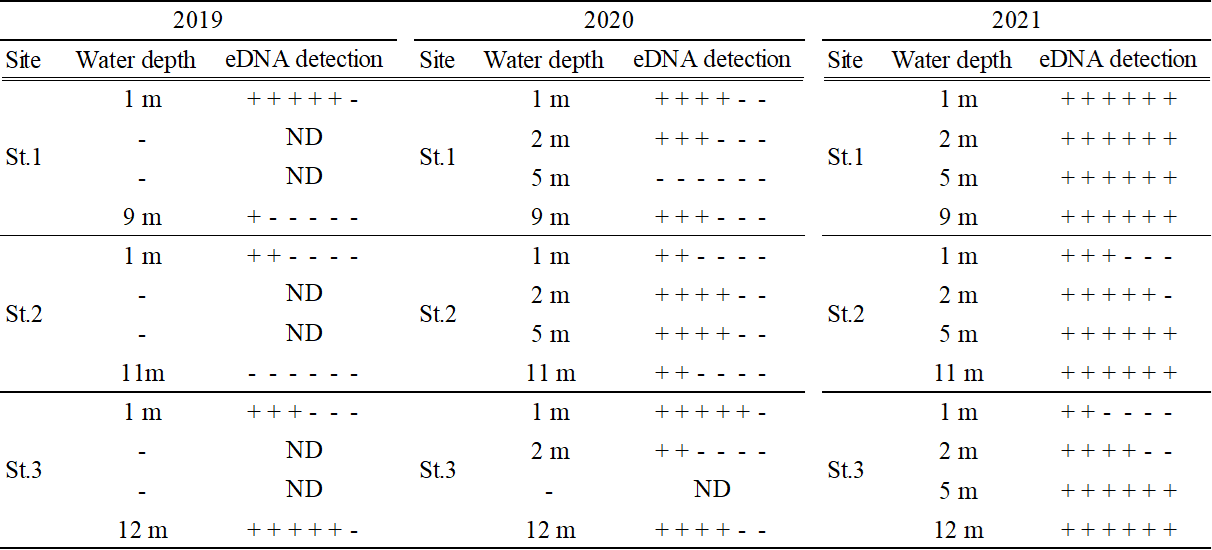


Table S9. Results of the GLMM for the relationship between the detection of masu salmon eDNA and the first and second components of PCA analysis (PC1, PC2) during lake stratification.


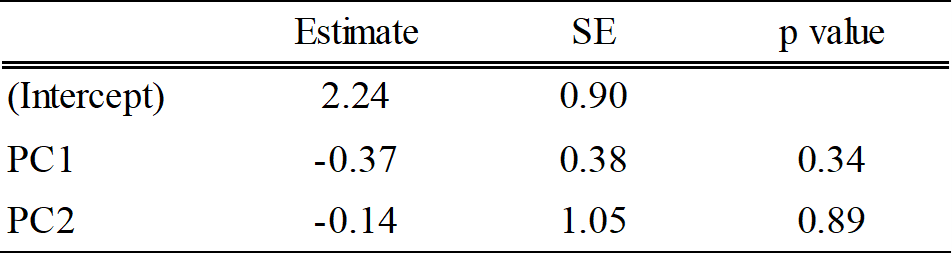


Figure legends

Fig. S1 Environmental data collected in Lake Yunoko, Japan, in August 2019.

Fig. S2 Environmental data collected in Lake Yunoko, Japan, in August 2020. Note that chlorophyll *a* in a water-column at 10- to 12-m depth could not be obtained due to the limited cable length of the handheld multiparameter meter (i.e., 10 m).

Fig. S3 Environmental data collected in Lake Yunoko, Japan, in November 2021.

Fig. S4 Vertical distribution of copy numbers (±SE) of masu salmon eDNA in the water column of Lake Yunoko in November 2021. Note that the scale of x-axis is different compared with Fig. 2 and Fig. 3 because of low DNA copy number of this species. The concentrations of masu salmon eDNA were below the detection limit in 2019 and 2020.

Fig. S5 Principal component analysis (PCA) plot of five environmental variables in the study sites. Vectors indicate the direction and strength of each environmental variable to the overall distribution.

Fig. S6 Five environmental variables which are deeper than 5m depth between stations in 2019 and 2020. Blue, brown, and green lines indicate the environmental variables in St.1, St.2 and St.3, respectively.

Fig. S7 The preferred water temperature of kokanee salmon (<13 °C, blue areas) and rainbow trout (<19 °C, yellow areas) in lake stratification periods.


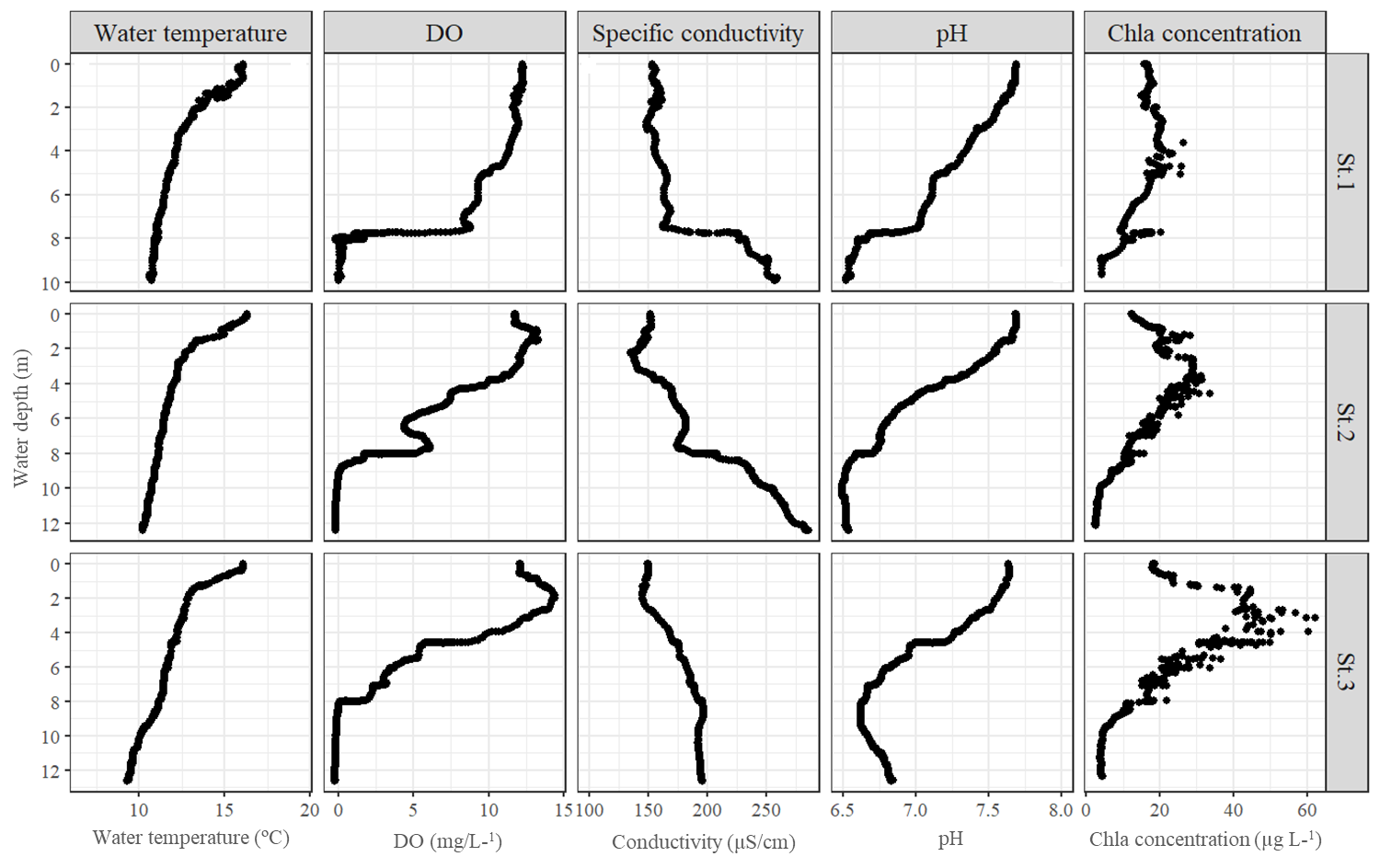


Figure S1


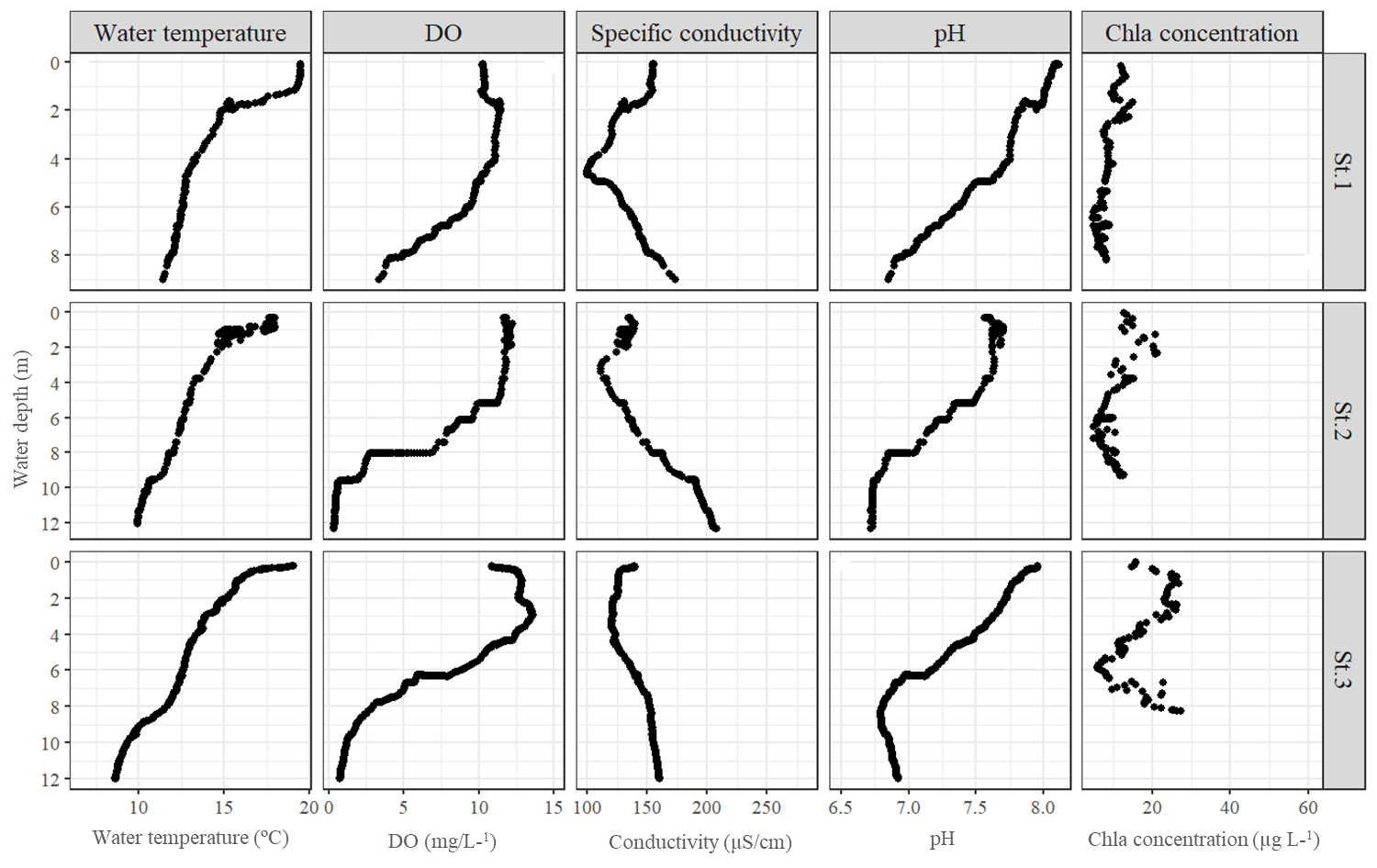


Figure S2


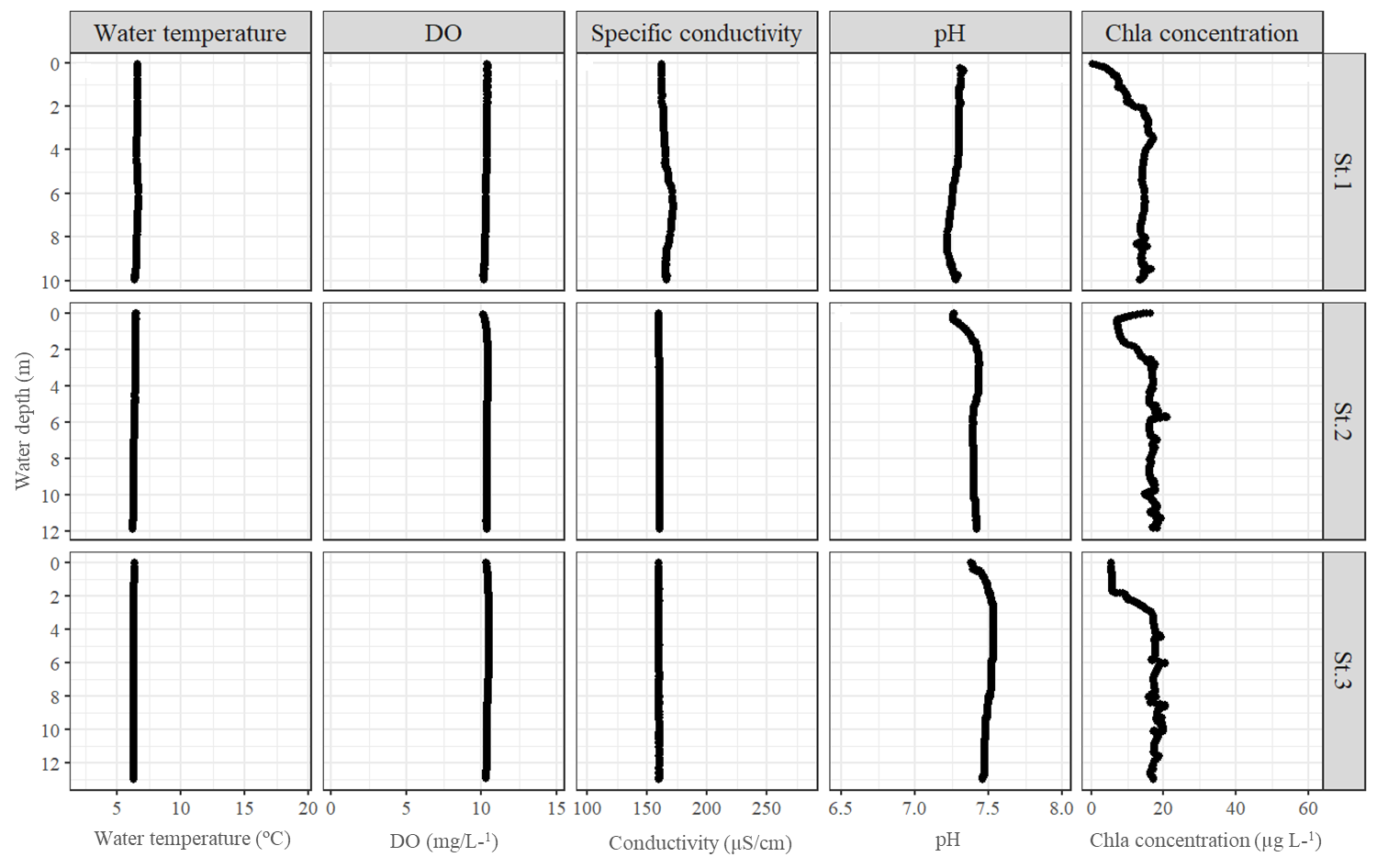


Figure S3


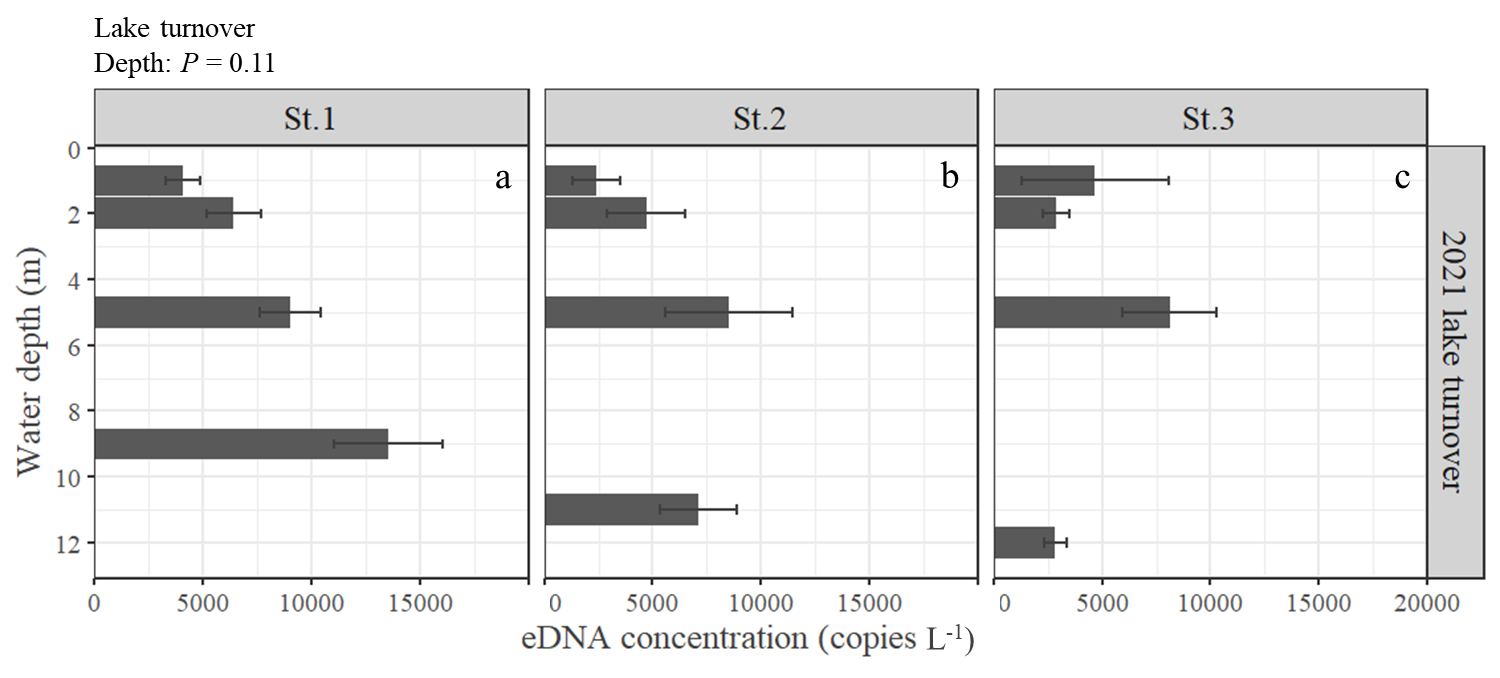


Figure S4


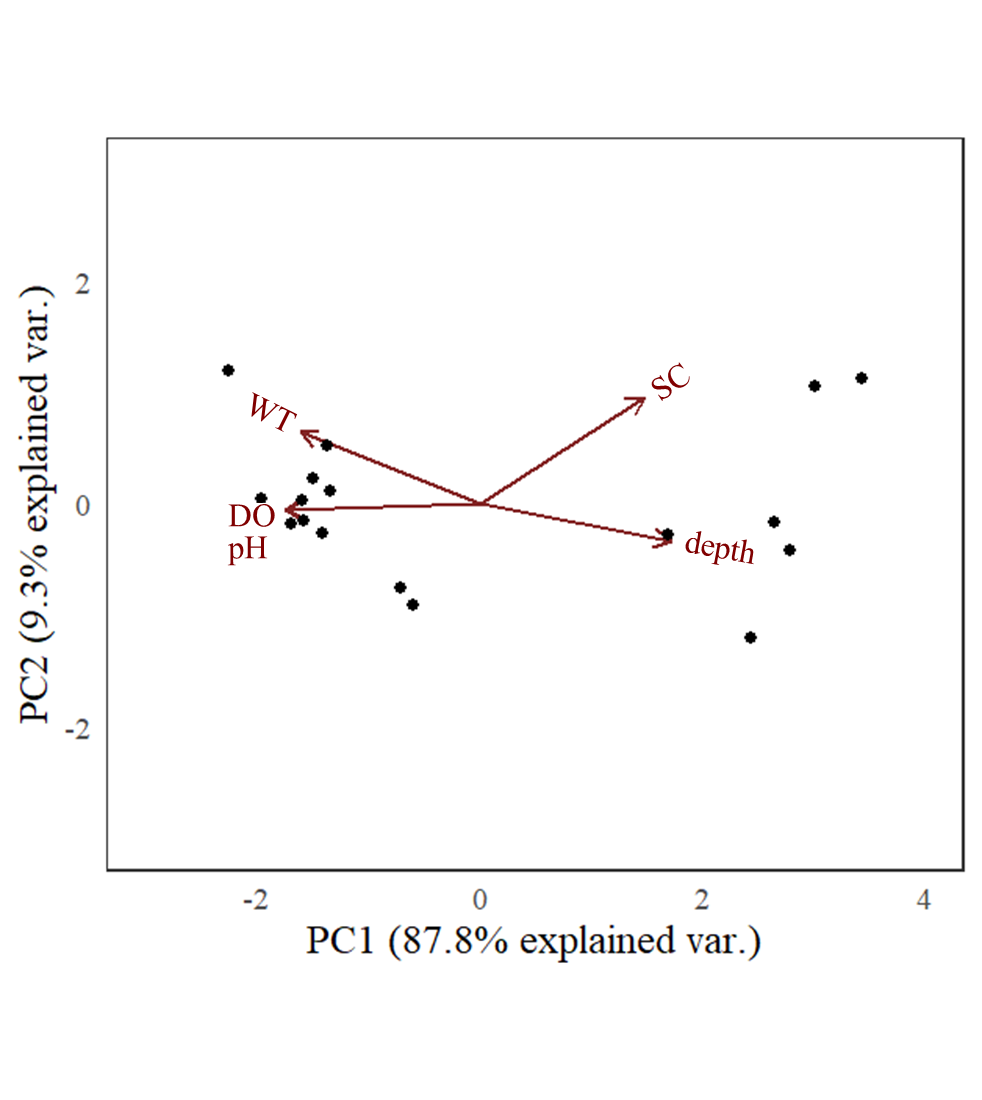


Figure S5


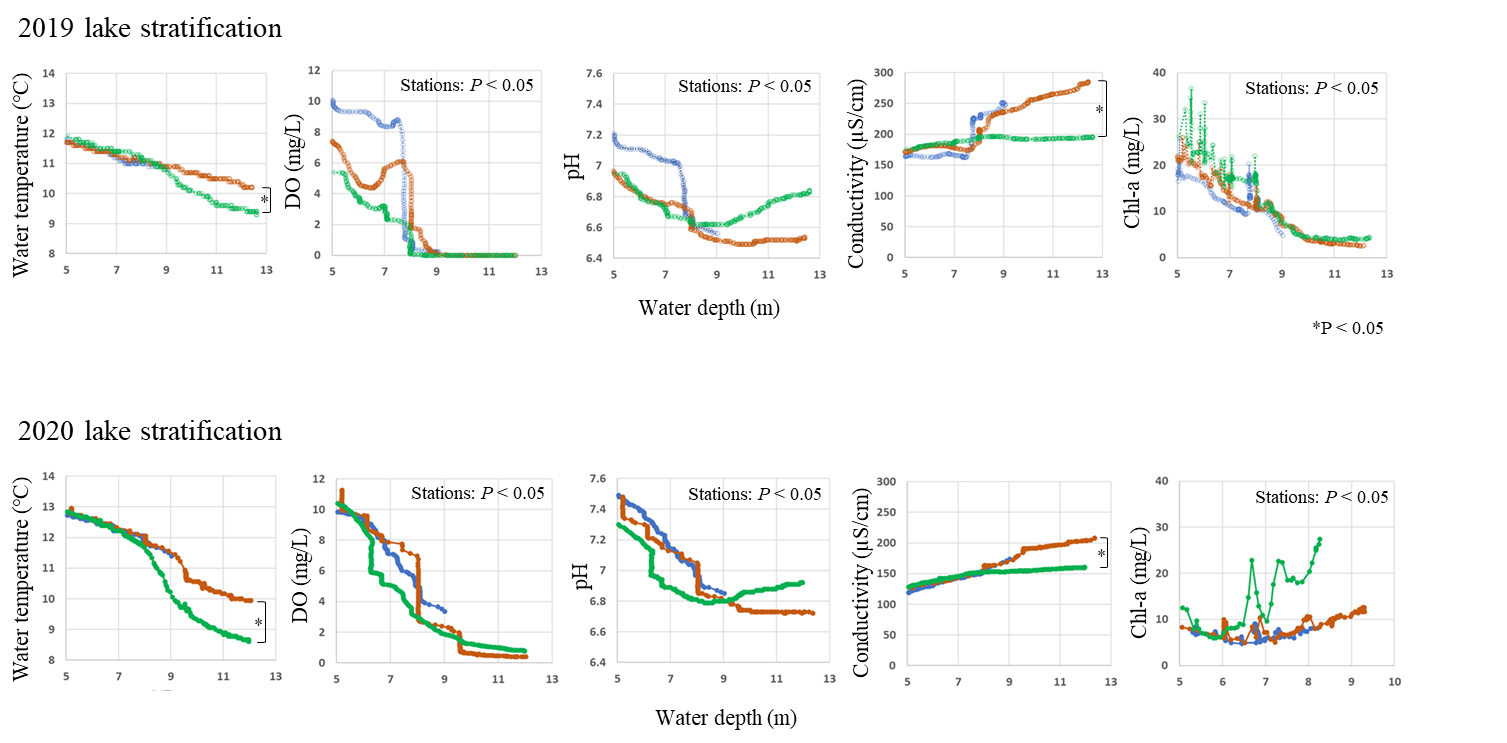


Figure S6


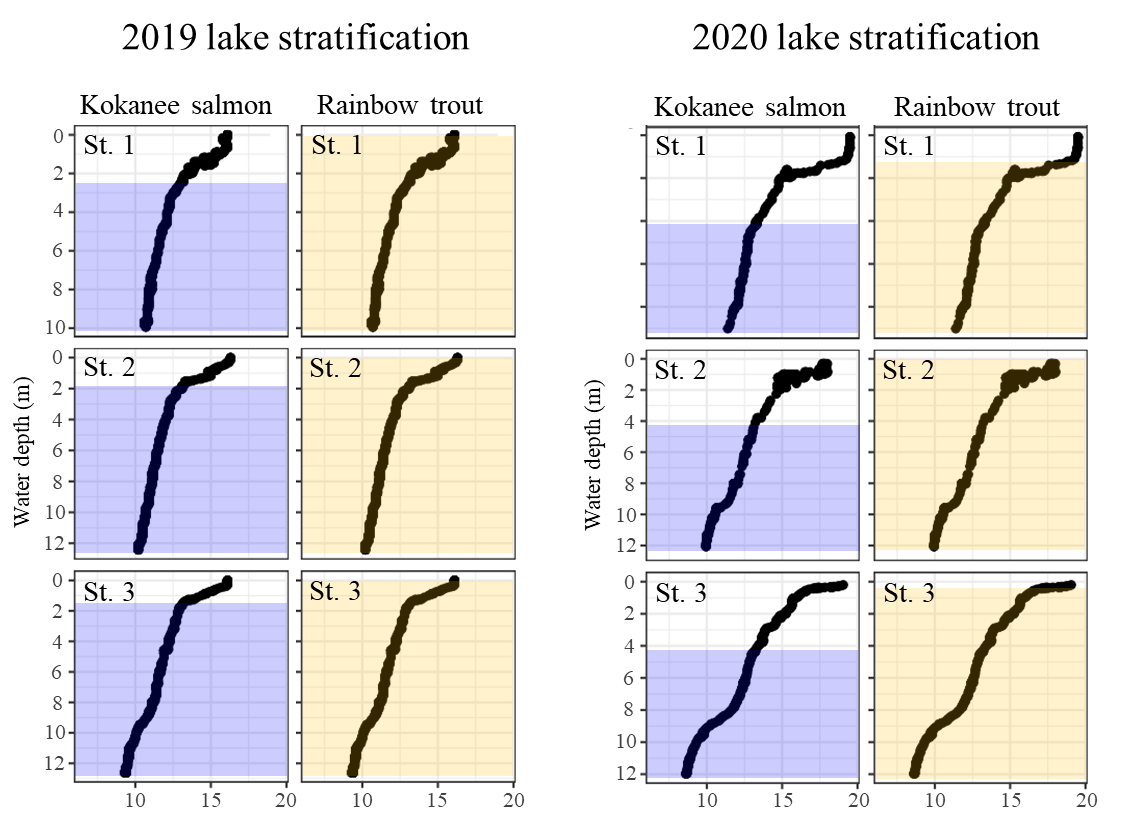


Figure S7
